# Composite grey matter fingerprints for genetic frontotemporal dementia

**DOI:** 10.1136/jnnp-2025-337186

**Published:** 2026-02-12

**Authors:** Arabella Bouzigues, Giulia Campana, Matthieu Joulot, Nicolas Gensollen, Lucy L Russell, Phoebe H Foster, Eve Ferry-Bolder, John Cornelis Van Swieten, Lize C Jiskoot, Harro Seelaar, Raquel Sánchez-Valle, Robert Laforce, Caroline Graff, Daniela Galimberti, Rik Vandenberghe, Alexandre de Mendonca, Pietro Tiraboschi, Isabel Santana, Alexander Gerhard, Johannes Levin, Sandro Sorbi, Markus Otto, Maxime Bertoux, Thibaud Lebouvier, Simon Ducharme, Chris Butler, Elizabeth Finger, Maria Carmela Tartaglia, Mario Masellis, James Benedict Rowe, Matthis Synofzik, Fermin Moreno, Barbara Borroni, Isabelle Leber, Gianluigi Zanusso, Jonathan Daniel Rohrer, Raffaella Migliaccio, on behalf of the GENetic Frontotemporal dementia Initiative (GENFI), Rhian Convery

**Affiliations:** 1 Hôpital Pitié-Salpêtrière, Paris Brain Institute, Paris, France; 2 Queen Square Institute of Neurology, University College London Dementia Research Centre, London, UK; 3 Department of Neurosciences, Biomedicine and Movement Sciences, University of Verona, Verona, Italy; 4 Neurology, Erasmus MC, Rotterdam, The Netherlands; 5 Department of Neurology, Hospital Clinic de Barcelona, Barcelona, Spain; 6 Institut d’Investigacions Biomèdiques August Pi i Sunyer, Barcelona, Spain; 7 Clinique Interdisciplinaire de Mémoire du CHU de Québec, Laval University, Québec City, Quebec, Canada; 8 Department of Neurobiology, Care Sciences and Society, KI-ADRC, KASPAC, Novum, Karolinska Universitetssjukhuset, Stockholm, Sweden; 9 Unit for Hereditary Dementias, Theme Inflammation and Aging, Karolinska Institute, Stockholm, Sweden; 10 Fondazione Ca’ Granda, IRCCS Ospedale Policlinico, Milan, Italy; 11 Centro Dino Ferrari, University of Milan, Milan, Italy; 12 Neurology Service, University Hospitals Leuven, Leuven, Belgium; 13 Leuven Brain Institute, KU Leuven, Leuven, Belgium; 14 Laboratory for Cognitive Neurology, Department of Neurosciences, KU Leuven, Leuven, Belgium; 15 Faculty of Medicine, University of Lisbon, Lisbon, Portugal; 16 Fondazione IRCCS Istituto Neurologico Carlo Besta, Milan, Italy; 17 Neurology Service, Faculty of Medicine, Coimbra University Hospital Centre, Coimbra, Portugal; 18 Faculty of Medicine, University of Coimbra Center for Neuroscience and Cell Biology, Coimbra, Portugal; 19 Division of Psychology Communication and Human Neuroscience, Wolfson Molecular Imaging Centre, The University of Manchester, Manchester, UK; 20 Department of Nuclear Medicine, Center for Translational Neuro- and Behavioral Sciences, University of Duisburg-Essen Faculty of Medicine, Essen, Germany; 21 Department of Geriatric Medicine, Klinikum Hochsauerland, Arnsberg, Germany; 22 Department of Neurology, Ludwig-Maximilians-Universität München, Munich, Germany; 23 German Centre for Neurodegenerative Diseases, Munich, Germany; 24 Munich Cluster of Systems Neurology (SyNergy), Munich, Germany; 25 Department of Neurofarba, University of Florence, Firenze, Italy; 26 IRCCS Firenze, Fondazione Don Carlo Gnocchi, Firenze, Italy; 27 Department of Neurology, University of Ulm, Ulm, Germany; 28 Neurology, Martin-Luther-Universitat Halle-Wittenberg, Halle (Saale), Germany; 29 Lille Neurosciences & Cognition U1172, Inserm, Université de Lille, CHU Lille, Lille, France; 30 Department of Psychiatry, Douglas Mental Health University Institute, Montreal, Quebec, Canada; 31 McConnell Brain Imaging Centre, Montreal Neurological Institute, Montreal, Quebec, Canada; 32 Nuffield Department of Clinical Neurosciences, Medical Sciences Division, University of Oxford, Oxford, UK; 33 Department of Brain Sciences, Imperial College London, London, UK; 34 Clinical Neurological Sciences, University of Western Ontario, London, Ontario, Canada; 35 Tanz Centre for Research in Neurodegenerative Disease, University of Toronto, Toronto, Ontario, Canada; 36 Sunnybrook Health Sciences Centre, Sunnybrook Research Institute, University of Toronto, Toronto, Ontario, Canada; 37 Department of Clinical Neurosciences, University of Cambridge, Cambridge, UK; 38 Division Translational Genomics of Neurodegenerative Diseases, Hertie-Institute for Clinical Brain Research and Center of Neurology, University of Tübingen, Tübingen, Germany; 39 Center for Neurodegenerative Diseases, DZNE, Tubingen, Germany; 40 Cognitive Disorders Unit, Department of Neurology, University Hospital of Donostia, San Sebastián, Spain; 41 Group of Neurodegenerative Diseases, Biogipuzkoa Health Research Institute, San Sebastián, Spain; 42 Molecular Markers Laboratory, IRCCS Istituto Centro San Giovanni di Dio Fatebenefratelli, Brescia, Italy; 43 Department of Clinical and Experimental Sciences, University of Brescia, Brescia, Italy; 44 Groupe Hospitalier Pitié-Salpêtrière, Department of Neurology, IM2A, AP-HP, Paris, France

**Keywords:** DEMENTIA, FRONTOTEMPORAL DEMENTIA, FRONTAL LOBE, IMAGE ANALYSIS, NEUROANATOMY

## Abstract

**Background:**

Brain structural changes in frontotemporal dementia (FTD) can occur decades before symptom onset. Precise characterisation of grey matter changes is necessary for developing models of biomarker progression, while better understanding the trajectory of the pathology is invaluable for prognosis and detecting treatment effects as we enter the era of clinical trials.

**Methods:**

Cortical and subcortical grey matter volume and thickness from structural MRI were assessed in a large cohort of 892 participants including presymptomatic and symptomatic carriers of mutations within the three main genetic causes of FTD (C9 open reading-frame 72 (C9orf72), progranulin (GRN) and microtubule-associated protein tau (MAPT)) compared with mutation-negative relatives (controls). We compared the distribution of grey matter changes of each metric at different stages of the disease cross sectionally. We aimed to identify grey matter composites for each genetic group which would show the earliest changes and which separated presymptomatic carriers from controls.

**Results:**

While C9orf72 mutation carriers showed widespread presymptomatic grey matter changes, MAPT and particularly GRN mutation carriers showed changes more proximally to symptom onset. Our composite grey matter signatures, which discriminate asymptomatic/prodromal carriers from controls with high to very high areas under the curve, involved bilateral thalami volumes, precuneus and postcentral thickness in C9orf72; left caudal middle frontal thickness, frontal pole and pars orbitalis volumes in GRN; right temporal pole volume and left insula thickness in MAPT mutation carriers.

**Conclusion:**

We propose the use of cortical thickness and volume measurements combined from multiple regions into a composite region of interest for each FTD genetic group to identify the earliest changes and track disease progression. Our quasi-longitudinal design illustrates that these regions continue to evolve throughout the symptomatic stages. Investigating how our selected composites progress and validating these in longitudinal samples will be invaluable for future clinical trials.

**WHAT IS ALREADY KNOWN ON THIS TOPIC**

- Structural brain changes in genetic frontotemporal dementia can be detected many years before clinical onset. However, the detailed evolution of whole-brain grey matter across disease stages and within different genetic groups remains understudied. Understanding early structural changes to use as sensitive early biomarkers is still needed to support prognosis, stratification and the measurement of treatment effects in clinical trials.

**WHAT THIS STUDY ADDS**

- This large cross-sectional study identifies gene-specific spatial signatures of early cortical and subcortical grey matter changes in C9orf72, GRN and MAPT mutation carriers. We highlight C9orf72 carriers’ diffuse presymptomatic atrophy and further emphasise that GRN and MAPT carriers’ structural changes align more with symptom onset. We derive composite grey matter metrics, using both volume and thickness of specific regions, that successfully distinguish presymptomatic carriers from controls with high accuracy and we illustrate their evolutive behaviour with disease progression as well as their potential to predict clinical decline. Moreover, we discuss the added value of using both cortical volume and thickness to disentangle neurodegenerative disease processes from possible neurodevelopmental differences, with GRN carriers showing a pattern dominated by degeneration, while C9orf72 carriers may show early structural differences shaped by both neurodevelopment and subsequent disease.

**HOW THIS STUDY MIGHT AFFECT RESEARCH, PRACTICE OR POLICY**

- These gene-specific composite biomarkers could accelerate the development and evaluation of targeted therapies by improving early detection, patient stratification and disease-tracking sensitivity in clinical trials. Validation in longitudinal cohorts may support future implementation of such structural composites as progression biomarkers in both research and clinical settings.

## Introduction

 Frontotemporal dementia (FTD) is a heterogeneous group of neurodegenerative diseases with up to 30% of cases caused by an autosomal dominant expansion or mutation in one of three main genes; C9 open reading frame 72 (C9orf72), progranulin (GRN) and microtubule-associated protein tau (MAPT).^1^ To ensure the success of therapeutic clinical trials in the genetic forms of FTD, research must highlight the most useful, reliable and valid biomarkers for trial outcome and progression monitoring.^2^ Recent research on the presymptomatic phase of genetic FTD, involving mutation carriers who are not yet showing clinical symptoms for a diagnosis, has demonstrated early grey matter atrophy.^3^,^7^ Such work highlights the potential of grey matter imaging, as a non-invasive biomarker, for the diagnosis and possibly prediction of clinical progression.

Moreover, investigating the early consequences of genetic mutations on the brain is crucial for understanding the trajectory of the associated pathology. Clinically, C9orf72 expansion carriers may present very early, non-evolving praxis impairment,^8^ cognitive disinhibition,^9^ verbal fluency changes^10^ and lifelong psychiatric vulnerabilities,^11^ alongside pronounced structural changes^3 7 12^ and possible brain functional connectivity deficits at the earliest stages.^13 14^ The precocity and focality of these symptoms suggest neurodevelopmental pathways as a possible origin. In this vein, a study in young presymptomatic carriers found that FTD mutations can cause structural brain effects detectable before the third decade of life.^15^ While young C9orf72 expansion carriers had smaller total intracranial brain volumes, young MAPT and GRN mutation carriers exhibited larger brain volumes, higher education levels and superior cognitive performance compared with non-carriers.^15^ These observations raise the possibility of a neurodevelopmental contribution, whereby certain FTD-associated mutations may exert advantageous effects during early life, at least in a subset of individuals.

Most studies in presymptomatic FTD have investigated either grey matter volume (surface or volume-based) or cortical thickness as markers for grey matter integrity. In healthy subjects, these measures are affected differently with ageing^16^ and explain independent fractions of variance in different cognitive scores.^17^ This is not surprising given that these features are controlled by different genetic factors and thus separate ontogenesis during brain development.^18^ Although these correlate greatly, studies have reported different patterns of changes when assessing these features in neurodegenerative diseases,^19 20^ including in genetic FTD.^5^ Thus, cortical thickness and volume measures are complementary for the investigation of grey matter changes, are affected differently by genetic mutations in FTD and may provide new insights on the relation between neurodegenerative and potential neurodevelopmental processes within these genetic groups.

In a large cohort of genetic FTD, we combined clinical phenotyping with neuroimaging to comprehensively chart the unfolding of early grey matter changes across genetic FTD. Using complementary surface-based volume and cortical thickness metrics, our work aimed to (1) provide a detailed characterisation of cortical and subcortical atrophy patterns, across the spectrum of disease from presymptomatic mutation carriers to fully symptomatic patients, identifying regions and metrics showing the earliest grey matter differences and which dissociate presymptomatic carriers from mutation-negative controls, (2) put forward a grey matter composite measure, corresponding to a combination of regions of interest (ROI) and brain metrics, specific for each genetic group, to define signatures of the earliest changes. Moreover, (3) we discuss the likely contribution of early neurodegeneration and neurodevelopment to the different grey matter changes reported, leveraging findings from the two grey matter measures studied here.

## Methods

### Participants

**Figure 1:**
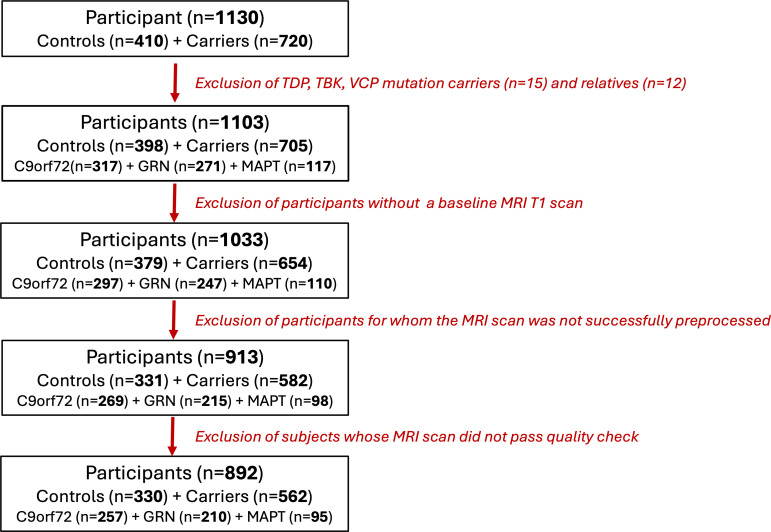
Flow chart showing the different steps for participant inclusion. MAPT, microtubule-associated protein tau. Sample sizes (n) at each stage are highlighted in bold.

At the time of the sixth data freeze in the GENFI study, 1130 participants had completed a baseline visit between January 2012 and January 2021 across 24 centres in the UK, Canada, Italy, Netherlands, Sweden, Portugal, Germany, France, Spain and Belgium. Participants were screened and genotyped at their local sites for the most common pathogenic genetic mutations for FTD. We excluded 27 participants who were carrying mutations in rarer FTD-causing genes including TBK1, TDBP and VCP. Of this cohort, we obtained 1033 participants with a volumetric T1 MRI scan acquired on one of six types of 3T scanners from three different vendors (Siemens Trio, Siemens Skyra, Siemens Prisma, Philips Achieva, Philips Ingenia, GE Discovery MR750). Specific acquisition parameters are reported in online supplemental Table 1. 141 scans were not successfully preprocessed, or participants were excluded as their scans did not pass quality assessments due to motion, incomplete spatial coverage or other imaging artefacts caused by pathologies unlikely to be attributed to FTD. The remaining 892 included participants were known to be either carriers of a pathogenic expansion in the C9orf72 gene (n=257) or of pathogenic mutations in GRN (n=210) or MAPT (n=95) genes, or mutation-negative first-degree relatives constituting the control group (n=330). Figure 1 presents a detailed flow chart of participant inclusion and exclusion.

All participants underwent a standardised clinical assessment as described previously,^3^ including the Clinical Dementia Rating Dementia Staging Instrument (CDR) with National Alzheimer Coordinating Centre Frontotemporal Lobar Degeneration component (CDR plus NACC FTLD) hereafter referred to as FTLD-CDR,^21^ a measure of disease severity from which a global score can be calculated. The global score was used in the present work to stage mutation carriers in each genetic group, with those having a score of 0 being considered asymptomatic, 0.5 as prodromal and 1, 2 or 3 representing symptomatic severity stages (table 1). We therefore had a control group and 15 gene/stage groups which are hereafter referred to as gene_stage (eg, C9orf72_1 for a C9orf72 carrier with an FTLD-CDR global score of 1).

**Table 1:** Sample size and demographic details for controls and mutation carriers further divided into genetic groups and disease stages according to the FTLD-CDR global score ranging from 0 (asymptomatic) to 0.5 (prodromal) and 1, 2, 3 (symptomatic)

Genetic group FTLD-CDR global		C9orf72					GRN					MAPT					Controls
		0	0.5	1	2	3	0	0.5	1	2	3	0	0.5	1	2	3	–
Sample	n	**128**	**46**	**21**	**32**	**30**	**131**	**32**	**23**	**11**	**13**	**49**	**17**	**10**	**10**	**9**	330
Gender	Females	75	28	6	10	10	88	15	14	3	9	30	10	5	2	3	189
	Males	53	18	15	22	20	43	17	9	8	4	19	7	5	8	6	141
Age	Mean	43.69	49.15	57.38	65.46	65.15	45.36	52.95	61.22	68.56	60.69	39.44	43.35	53.61	56.93	57.03	45.6
	SD	11.36	12.29	12.43	6.97	7.88	11.4	12.46	8.09	7.19	6.15	10.78	12.46	9.26	10.59	9.08	12.91
Education	Mean	14.23	14.22	13.29	13.56	12.1	14.89	13.83	12.83	11.64	10.54	14.45	14.06	13.2	13.4	14.33	14.5
	SD	3.14	2.59	3.85	3.82	4.23	3.68	4.15	3.55	3.72	2.96	3.32	2.56	3.71	2.07	4.39	3.43

C9orf72, C9 open reading-frame 72; FTLD-CDR, Clinical Dementia Rating Dementia Staging Instrument (CDR) with National Alzheimer Coordinating Centre Frontotemporal Lobar Degeneration component (CDR plus NACC FTLD); GRN, progranulin; MAPT, microtubule-associated protein tau.

### Clinical and cognitive measures

The GENFI study includes extensive clinical and neuropsychological assessments which were completed within 3 months of MRI acquisition.

The FTLD-CDR is an eight-domain rating scale involving cognition, language and behaviour, based on informant reports and allows for the assessment of these core clinical features of FTD. This scale enables the calculation of a ‘sum-of-boxes’ score (SOB) ranging from 0 to 24 and a global score ranging from 0 to 3 (0, 0.5, 1, 2, 3). The scoring rules for determining the global score are described in the original paper and were used in this study to stratify carriers into asymptomatic, prodromal and symptomatic stages.^21^

### T1 scan processing

T1-weighted scans were processed using FreeSurfer V.7.1.1. FreeSurfer is a surface-based analysis (SBA) meaning that for each vertex, volume is the product of grey matter area and cortical thickness.^22^ In comparison to voxel-based morphometry, SBA presents the advantage of not requiring that the images be aligned to a common space, which, together with the segmentation method, has the potential for introducing additional confoundings, such as misalignment, misclassification, as well as false detection of normal variants of folding patterns as focal changes on grey matter volume. Cortical thickness is calculated as the mean distance between vertices of a corrected, triangulated, estimated grey/white matter surface and grey matter/cerebrospinal fluid (pial) surface.

Thus, grey-matter volumes and cortical thickness values were generated using the recon-all cross-sectional approach^23^ which generated values across 327 684 vertices of the cortex, with a full-width-half-maximum of 20. This pipeline performs cortical surface extraction, segmentation of subcortical structures, cortical thickness estimation, spatial normalisation onto the FreeSurfer surface template (FsAverage), and parcellation of cortical regions based on different atlases. We ran this using Clinica’s t1-freesurfer pipeline.^24^

FreeSurfer segmentation outputs were each visually inspected for severe errors. When severe errors occurred, or when FreeSurfer crashed without providing outputs and this was not fixed by repeated attempts, FreeSurfer analyses of those scans were omitted (n=120). No manual correction of FreeSurfer segmentations was performed.

### Statistical analyses

Statistical analyses were performed using BrainStat for neuroimaging group comparisons, STATA V.16.0 and R V.4.3.3 for demographic and clinical analyses. The significance level was set at p<0.05 unless otherwise stated.

### Participant demographics and grey matter in controls

To assess demographic differences between participants from different genetic groups, we compared participant demographics from the same severity stage between genetic groups and with controls using bootstrapped linear regression.

To assess whether mean cortical thickness and total volume were related to age, sex and education in controls, we assessed the correlation between continuous variables using Spearman rank correlations with 95% confidence limits and assessed differences between males and females using Kruskal-Wallis χ² tests.

### Grey matter vertex-wise group differences

We compared cortical thickness and volume values between each gene_stage group and controls using separate general linear models per cortical vertex and subcortical structure. These models were adjusted for participants’ age, sex, education, total intracranial volume and site of data acquisition. Pointwise false positives were controlled for with false-discovery rate to account for the 327 684 vertices/12 subcortical structures and cluster-level multiple comparisons were controlled for with random-field theory, applying a vertex-wise cluster threshold of 0.01. Moreover, as the same control group was used in fifteen models, we corrected the p value threshold using Bonferroni (0.05/15=p<0.003). We thus identified regions which showed significant reductions of grey matter volume, cortical thickness or both in each gene_stage group compared with controls. We calculated effect sizes for each region using Cohen’s d.

### Receiver operating characteristic curve analysis

We identified clusters from the vertex-wise group differences analyses which showed early volume or cortical thickness reductions. Those with the highest effect sizes within each genetic group were mapped to ROIs from the Desikan atlas.^25^ These individually selected ROIs involved bilateral thalamus volume, bilateral precuneus thickness and bilateral postcentral gyrus thickness for C9orf72 carriers, left frontal pole volume, pars orbitalis volume and caudal middle frontal thickness for GRN carriers and right temporal pole volume and left insula thickness for MAPT carriers. For each genetic group, such ROIs were included together in receiver operating characteristic curve (ROC) computations, standardising and splitting the data into training and testing subsets, allocating 70% for training and 30% for testing, while ensuring randomisation and reproducibility through a fixed random seed. A logistic regression classifier was implemented allowing for up to 1000 iterations to ensure convergence. The area under the ROC curve was calculated to quantify each composite’s ability to distinguish between classes, separating asymptomatic (FTLD-CDR global=0) and prodromal (FTLD-CDR=0.5) carriers from controls.

### Quasi-longitudinal progression estimation of selected grey matter ROIs

To estimate the progression of the different selected ROIs, for each genetic group we calculated carriers’ z-scores for each ROI based on controls’ mean and SD for that specific ROI. We then plotted these for each FTLD-CDR group in our quasi-longitudinal design.

### Clinical validity of selected grey matter ROIs

To assess the clinical validity of our imaging grey-matter fingerprints, we correlated these with the FTLD-CDR sum of boxes (SOB) scores using Spearman correlations in presymptomatic carriers. To assess the predictive validity of our marker, we also assessed whether our early grey-matter fingerprints at baseline visit were correlated with clinical change assessed at the subsequent follow-up visit carried out on average 15.4 months after the baseline for C9orf72 carriers, 18.1 for GRN carriers and 15.3 for MAPT carriers. Only carriers with an FTLD-CDR score of 0.5 were included in these analyses as many of those with a score of 0 did not show much variability across clinical and neuroimaging measures. Moreover, some carriers with an FTLD-CDR global score of 0.5 had already received a clinical diagnosis, reflecting the fact that there is not always perfect correspondence between the FTLD-CDR global score and clinicians’ judgement. To ensure that the correlations between grey matter measures and clinical severity in presymptomatic carriers were not biased by these individuals, who are likely to exhibit greater atrophy, we excluded them from the analyses (11 C9orf72, 5 GRN and 3 MAPT) but also report the results when including them. As some subjects only had a baseline visit, correlations with follow-up scores were performed on reduced sample sizes (C9orf72 n=11; GRN n=12; MAPT n=8).

### Data availability

Data will be shared according to the GENFI data sharing agreement, after review by the GENFI data access committee with final approval granted by the GENFI steering committee.

## Results

### Participant demographics and grey matter in controls

All symptomatic groups (apart from MAPT_1), as well as GRN_0.5 and MAPT_0.5, were significantly older than controls. Moreover, MAPT_0 was, on average, significantly younger than controls. C9orf72_1 and C9orf72_2 had a significantly different ratio of males to females, with more males, compared with controls. The ratio of males to females was significantly different in C9orf72_1 and C9orf72_2 groups compared with controls; they included more males than the control group did. Finally, C9orf72_3, GRN_2, GRN_3 and MAPT_2 had significantly fewer years of education compared with controls. These results, as well as significant differences between different genetic groups of the same severity stage, are summarised in online supplemental Table 2. The distribution of age and education within each group is available in online supplemental Figure 1.

In controls, total grey matter volume and average cortical thickness were significantly negatively correlated with age (r=−0.34, p<0.0001; r=−0.45; p<0.0001) and positively correlated with education (r=0.21, p=0.0002; r=0.13, p=0.02). Moreover, male control participants had significantly higher total grey matter volumes compared with females (p<0.0001), but no differences were observed for average cortical thickness. Finally, total grey matter volume and average cortical thickness differed according to site of acquisition (p=0.04; p=0.0006). These relationships are summarised in online supplemental Figure 2.

### Grey matter vertex-wise group differences

**Figure 2:**
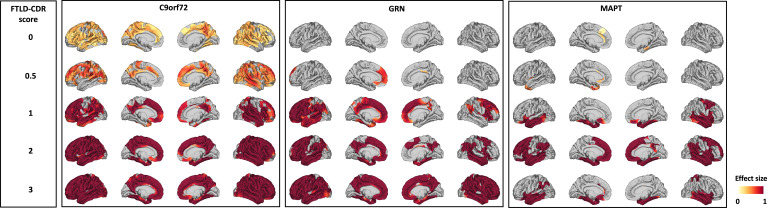
Cortical thickness effect sizes (heat map represents Cohen’s d) for each FTD gene group and for varying levels of disease severity according to the FTLD-CDR score, compared with controls. p<0.003, Bonferroni corrected for multiple comparisons, cluster threshold=0.01, FDR corrected, model adjusted for age, sex, years of education, site of acquisition and total intracranial volume. FDR, false-discovery rate; FTD, frontotemporal dementia; C9orf72, C9 open reading-frame 72; GRN, progranulin; MAPT, microtubule-associated protein tau; FTLD-CDR, Clinical Dementia Rating Dementia Staging Instrument (CDR) with National Alzheimer Coordinating Centre Frontotemporal Lobar Degeneration component (CDR plus NACC FTLD).

**Figure 3:**
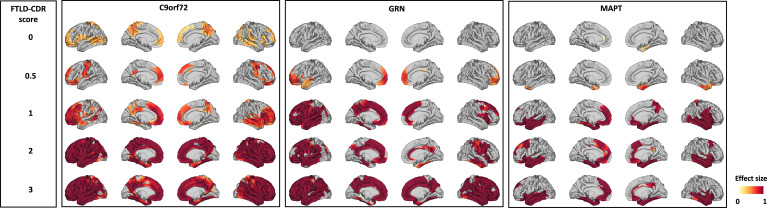
Cortical volume effect sizes (heat map represents Cohen’s d) for each FTD gene group and for varying levels of disease severity according to the FTLD-CDR score, compared with controls. p<0.003, Bonferroni corrected for multiple comparisons, cluster threshold=0.01, FDR corrected, model adjusted for age, sex, years of education, site of acquisition and total intracranial volume. FDR, false-discovery rate; FTD, frontotemporal dementia; C9orf72, C9 open reading-frame 72; GRN, progranulin; MAPT, microtubule-associated protein tau; FTLD-CDR, Clinical Dementia Rating Dementia Staging Instrument (CDR) with National Alzheimer Coordinating Centre Frontotemporal Lobar Degeneration component (CDR plus NACC FTLD).

Significant group differences compared with controls are presented as effect sizes on the cortical surface in figure 2 (cortical thickness) and figure 3 (volume). A combination of these is presented in online supplemental Figure 3.

From the asymptomatic stage (FTLD-CDR=0), C9orf72 carriers showed significant reductions in grey matter volume and cortical thickness within several cortical regions as well as left amygdala, bilateral thalamus and hippocampus, before spreading to affect the entirety of frontal, temporal and parietal lobes as well as most subcortical structures at the most severe symptomatic stages (FTLD-CDR=2/3). Specifically, at the asymptomatic stage, bilateral thalamus volume and bilateral precuneus thickness showed the highest effect sizes. From the prodromal stages, orbitofrontal cortex and superior frontal gyrus presented cortical thickness and volume loss bilaterally as well as precentral and postcentral gyri involvement. More posteriorly, specific cortical thickness reduction was observed in temporoparietal (supramarginal gyrus, mainly left-sided) and temporooccipital regions (bilaterally). High effect sizes were notable for bilateral postcentral gyrus thickness. With disease progression to symptomatic stages, volume and thickness overlapped greatly in C9orf72 carriers.

GRN carriers did not differ significantly from controls at the asymptomatic stage for any cortical or subcortical grey matter region volume or thickness. Carriers started showing volume loss from the prodromal stage (FTLD-CDR=0.5) within orbitofrontal cortex as well as the most caudal part of left middle frontal gyri. Highest effect sizes were noticed within left frontal pole and pars orbitalis volumes as well as left caudal middle frontal gyrus (Brodmann areas 6 and 8) thickness. Fully symptomatic carriers (from FTLD-CDR=1) showed volume reductions and cortical thinning of the entire frontal, temporal and parietal regions, particularly within inferior frontal gyrus (pars orbitalis, pars triangularis and pars opercularis) and progressively affecting the frontomesial regions and the temporoparietal junction, initially of the left hemisphere and then bilaterally. Several subcortical structures showed significantly reduced volume from symptom onset.

MAPT asymptomatic carriers (FTLD-CDR=0) showed some significant cortical thickness reductions compared to controls within left anterior cingulate cortex and right medial temporal volume reductions and thinning, but effect sizes remained low. From the prodromal stage (FTLD-CDR=0.5), notable right temporal pole volume showed high effect sizes as well as left insula thickness. Bilateral entire temporal lobe atrophy was notable at the earliest symptomatic stages (FTLD-CDR=1) as well as right parietal lobe thinning, medial frontal and parietal volume reductions. Bilateral frontal lobe atrophy (particularly thinning) was widespread from later stages. Several subcortical structures showed significantly reduced volume from symptom onset.

The statistical results for cortical volume, cortical thickness and subcortical volume group differences compared with controls can be found in online supplemental Tables 3–5, respectively.

Regions and metrics with the highest effect sizes for each group at the asymptomatic or prodromal stages were identified and mapped to the Desikan atlas ROIs to form a composite. These are summarised in table 2.

**Table 2:** Selected ROIs and metrics for each group were identified based on the largest effect sizes from the vertex-wise analyses and subsequently mapped onto Desikan atlas regions.

		C9orf72	GRN	MAPT
ROIs	Volume	Bilateral thalamus	Left frontal pole Left pars orbitalis	Right temporal pole
	Thickness	Bilateral precuneus Bilateral postcentral gyrus	Left caudal middle frontal gyrus	Left insula

These regions showed reductions in volume or cortical thickness compared with controls in early disease severity groups (ie, individuals with an FTLD-CDR global score of 0 or 0.5).

C9orf72, C9 open reading-frame 72; FTLD-CDR, Clinical Dementia Rating Dementia Staging Instrument (CDR) with National Alzheimer Coordinating Centre Frontotemporal Lobar Degeneration component (CDR plus NACC FTLD); GRN, progranulin; MAPT, microtubule-associated protein tau; ROI, region of interest.

### ROC analysis

**Figure 4:**
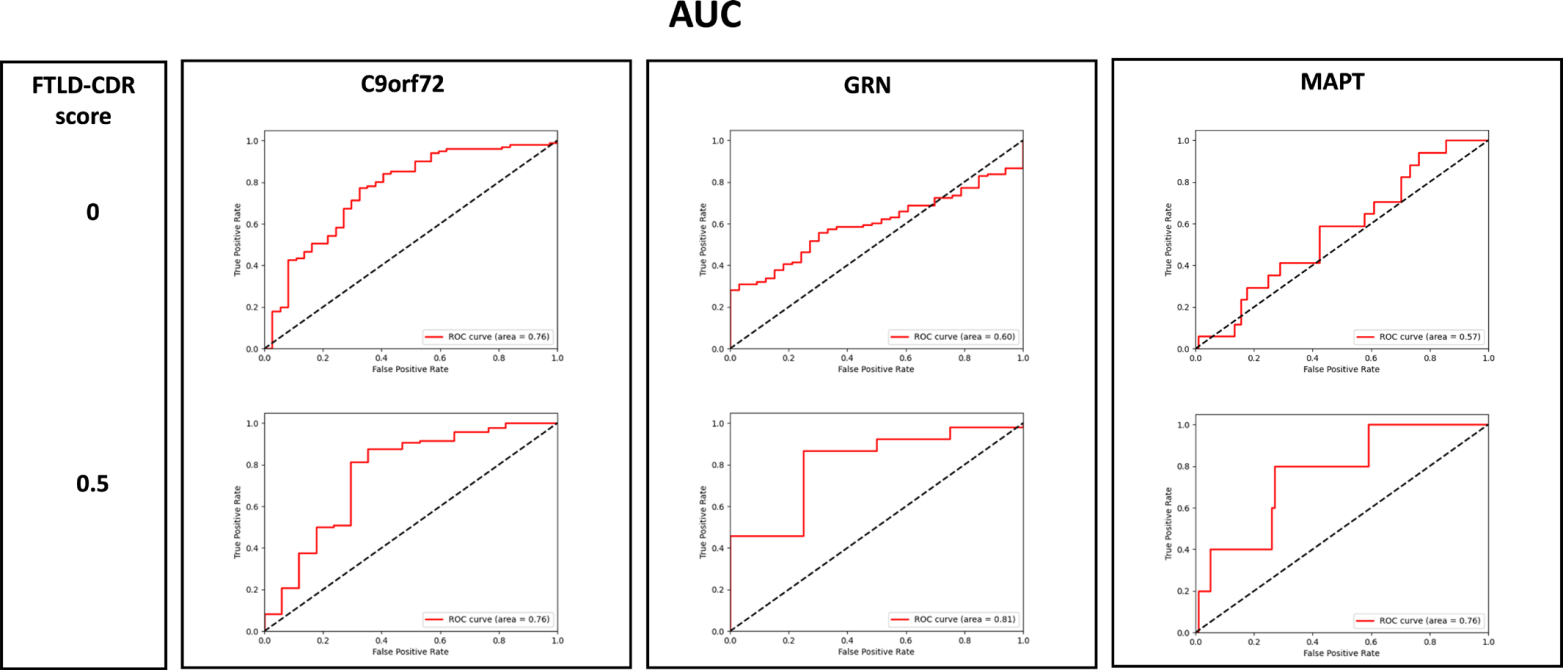
Area under the receiver operating characteristic (ROC) curve for each combination of ROIs and metrics for each gene_stage group. AUC, area under the curve; MAPT, microtubule-associated protein tau; ROI, region of interest; FTLD-CDR, Clinical Dementia Rating Dementia Staging Instrument (CDR) with National Alzheimer Coordinating Centre Frontotemporal Lobar Degeneration component (CDR plus NACC FTLD).

The clinical usefulness of the combination of the identified regions and metrics for each group was assessed with a ROC analysis. This showed moderate to high areas under the curve (AUC) in FTLD-CDR 0 groups (C9orf72: 0.76; GRN: 0.60; MAPT: 0.57). In FTLD-CDR 0.5 groups, AUCs were high to very high (C9orf72: 0.76; GRN: 0.81; MAPT: 0.76) (figure 4).

### Quasi-longitudinal progression estimation of selected composites

**Figure 5:**
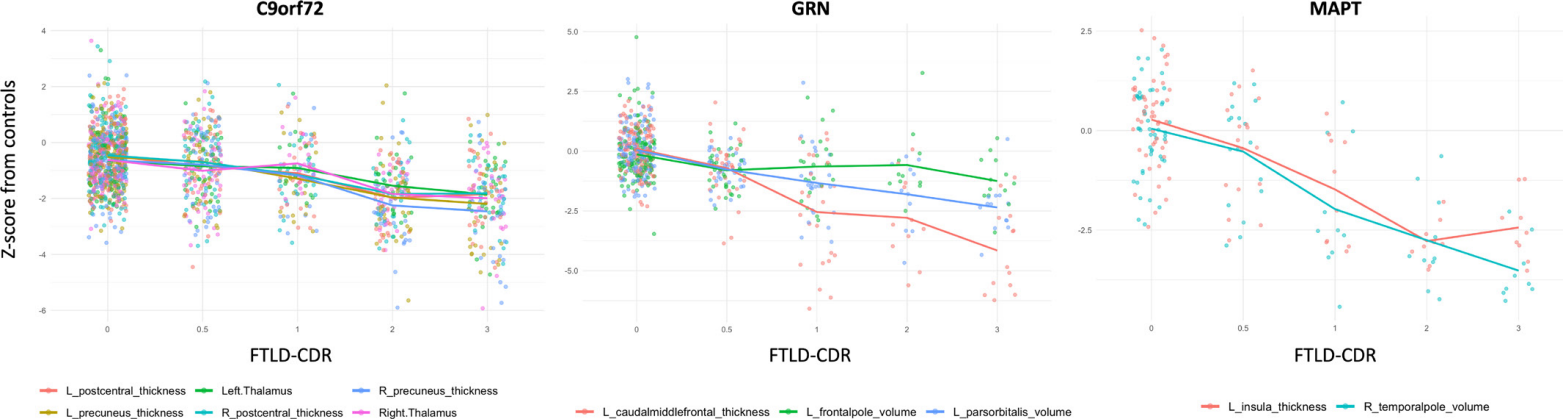
Estimated progression of grey matter from each ROI in each genetic group based on cross-sectional cohort. FTLD-CDR, Clinical Dementia Rating Dementia Staging Instrument (CDR) with National Alzheimer Coordinating Centre Frontotemporal Lobar Degeneration component (CDR plus NACC FTLD); C9orf72, C9 open reading-frame 72; GRN, progranulin; MAPT, microtubule-associated protein tau; ROI, region of interest.

We found that grey matter for all ROIs from our composites decreased over time, with the ROIs showing the biggest differences reaching 3 SDs from controls at the latest stage of the disease (FTLD-CDR=3). Figure 5 presents our estimated progression of each ROI in this quasi-longitudinal design.

### Clinical validity of selected composites

**Figure 6:**
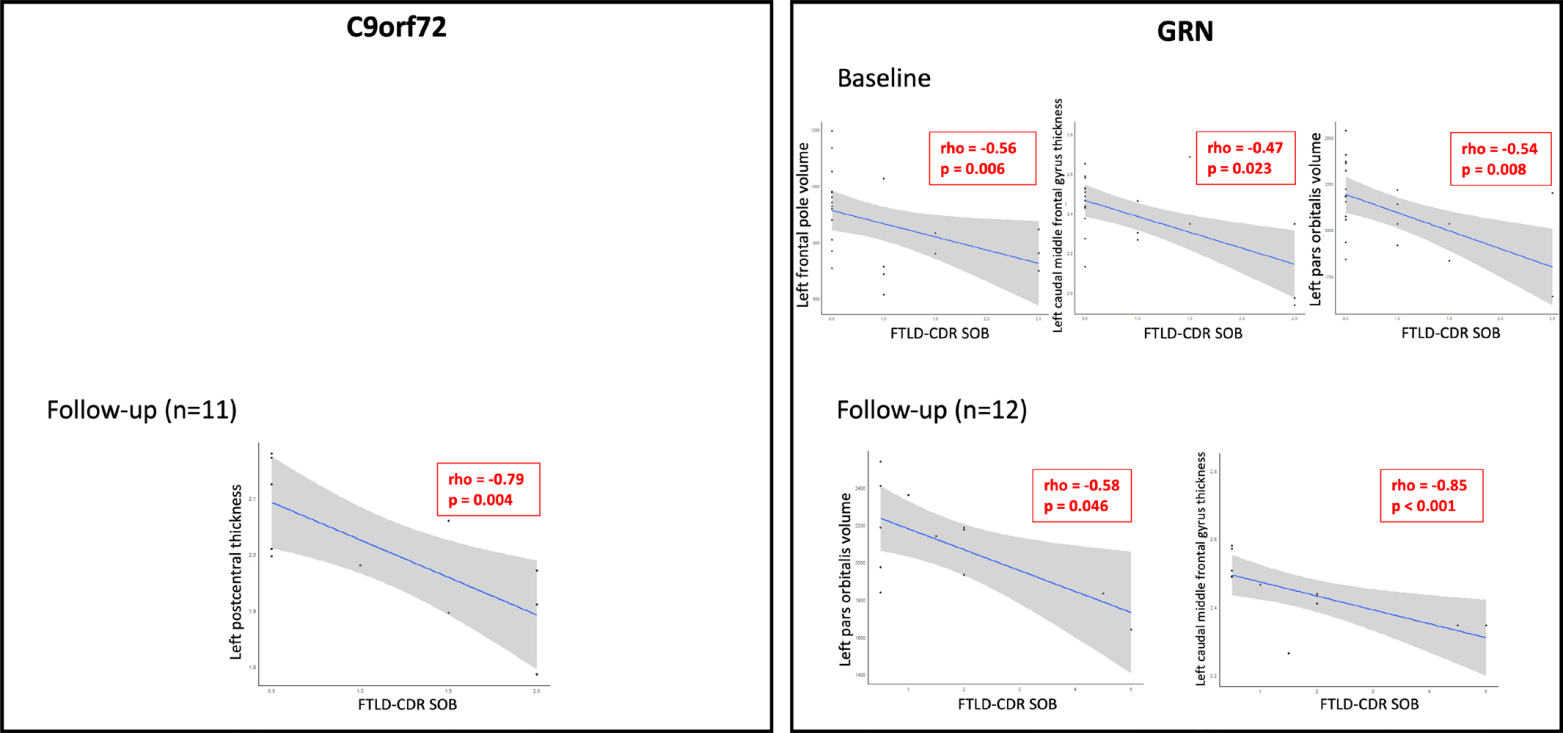
Correlations between grey matter signatures and clinical severity as well as clinical progression in presymptomatic carriers. FTLD-CDR, Clinical Dementia Rating Dementia Staging Instrument (CDR) with National Alzheimer Coordinating Centre Frontotemporal Lobar Degeneration component (CDR plus NACC FTLD); SOB, sum-of-boxes; C9orf72, C9 open reading-frame 72; GRN, progranulin.

In C9orf72 carriers, left postcentral gyrus thickness significantly predicted follow-up FTLD-CDR SOB scores (rho=−0.79, p=0.004). In GRN carriers, all three grey matter regions (left frontal pole volume, left pars orbitalis volume and left caudal middle frontal thickness) were significantly correlated with baseline FTLD-CDR SOB scores (r=−0.56, p=0.006; r=−0.54, p=0.008; r=−0.47, p=0.023, respectively). Moreover, left pars orbitalis volume and left caudal middle frontal thickness were significant predictors of follow-up FTLD-CDR SOB scores (r=−0.58, p=0.046; r=−0.85, p<0.001, respectively). Finally, no grey matter regions were significantly correlated with clinical severity or clinical progression in MAPT carriers. Significant results are presented in figure 6. As expected, when including carriers who already had a diagnosis, we found some more significant correlations in C9orf72 and MAPT carriers. Namely, in C9orf72 carriers, we found a significant correlation between right precuneus thickness and baseline and follow-up FTLD-CDR SOB scores. In MAPT carriers, we found that left insula volume significantly predicted follow-up FTLD-CDR SOB scores. These results are presented in online supplemental Figure 4.

## Discussion

### Summary of findings

Our study had two main objectives. First, we aimed to define the distribution of grey matter loss in the three most common forms of genetic FTD by measuring volume and cortical thickness across the spectrum of disease severity in a large sample. Second, we wished to select the most appropriate combination of grey matter ROIs and MRI metrics to define signatures of the earliest grey matter changes for each genetic group.

Overall, we found that C9orf72 expansion carriers showed the earliest grey matter changes from the asymptomatic stage of the disease, while GRN mutation carriers had little to no evidence of such structural changes until symptom onset. MAPT carriers showed some focal early signs of change. Although there was great overlap between volume and thickness changes in all groups, our results show some temporal and spatial discrepancies which we discuss below, and which highlight the usefulness of considering both metrics when validating future grey matter biomarkers.

The identified grey matter signatures were useful for discriminating presymptomatic individuals from controls with good accuracy, particularly in prodromal individuals. When further validating the usefulness of these grey matter composites for predicting disease severity and progression, we highlight their use in GRN but not so much in C9orf72 and MAPT mutation carriers. These final analyses are nonetheless to be interpreted with caution in view of the very limited samples available. We suggest these composite grey matter signatures as early disease biomarkers suitable for both therapeutic trials and as future diagnostic tools in genetic FTD.

### Early grey matter changes are specific to each genetic group

C9orf72 expansion carriers demonstrated extremely widespread cortical thinning across the cortex, even in the asymptomatic phase when no major behavioural, language or cognitive changes are identified. Prominent volume reductions were also observed within frontal, medial parietal and subcortical regions. Our findings align with previous studies showing early reductions in these areas^3^,^57 8 26 27^ but extend this to the largest sample of asymptomatic carriers to date (n=128), determined using a reliable clinical severity scale and using a whole-brain vertex-wise approach without relying on predefined parcellations. While earlier studies suggested that some changes, like thalami volume reductions, occur up to 25 years before symptom onset,^3^ such estimates based on mean family age of onset have since been shown to be unreliable for C9orf72 expansion carriers.^28^ However, findings in a young subgroup of this cohort (aged between 19 and 29) indicate that thalamic reductions may emerge decades before onset,^15^ as also already shown before.^8^ Although grey matter reductions were widespread in our study, effect sizes were generally low in asymptomatic C9orf72 carriers, likely reflecting variability in subsequent symptomatic presentations of such mutation carriers, including behavioural, motor (Amyotrophic Lateral Sclerosis (ALS)), neuropsychiatric or even memory deficits.^29^ Early grey matter changes may hold potential for predicting clinical phenotypes at the individual level,^30^ warranting further longitudinal studies with converting individuals.

In contrast, GRN mutation carriers showed no grey matter changes in the asymptomatic phase (n=131), consistent with previous findings.^45 7 31^,^34^ Some studies have reported early changes in presymptomatic GRN carriers,^3 4^ but these findings often involved small samples or did not survive correction for multiple comparisons. By using the FTLD-CDR score, we better characterised clinical stages such as the dissociation between asymptomatic and prodromal carriers. We reveal significant volume reductions in left orbitofrontal regions, with high effect sizes, at the prodromal phase. A recent study highlights frontal lobe regions specifically show age-related reductions in GRN carriers.^34^ By the symptomatic stage, striking grey matter reductions span most left frontal, temporal and parietal lobes, mirroring rapid cognitive decline and NfL increases during transition from presymptomatic to symptomatic stage.^35^,^37^

Asymmetrical patterns of atrophy have been previously reported in symptomatic GRN carriers, either left-sided or right-sided.^438^,^41^ Asymmetrical left-sided atrophy was notable in our prodromal GRN carriers. There are several reasons why we may find this left-sided asymmetry. First, it may be that a group-wise analysis obscures more subtle, or seen in fewer individuals, right-sided grey matter reductions. Second, it may be due to a sampling bias, with many more prodromal carriers who will go on to develop a primary progressive aphasia (PPA) in this specific cohort. Interestingly, the asymmetrical left-sided atrophy we identified was maintained at the earliest symptomatic stage, which is in line with the fact that over 50% of the GRN patient cohort in our sample had a diagnosis of PPA. A recent study has suggested that left-sided GRN carriers may show a faster rate of subsequent impairment.^41^ Taken together, these results highlight that left-sided early regions of grey matter atrophy may be of particular importance for monitoring progression in GRN carriers.

Finally, in MAPT mutation carriers, we observed very focal grey matter reductions in anterior temporal lobe and left insula, emerging possibly in the asymptomatic phase but confirmed at the prodromal stage. Symptomatic MAPT carriers showed atrophy extending to medial and superior frontal lobes and precuneus, with further posterior expansion in later stages. These patterns align with previous work,^3 4 7 31 42^ with our study providing results from the largest presymptomatic cohort to date. It remains unclear if there is an asymmetrical pattern, with only volume changes on the right, highlighting the complementarity of exploring both volume and thickness measures.^5^ Moreover, as in GRN carriers, a left-sided atrophy pattern may be predictive of a future PPA clinical phenotype, particularly in view of the early temporal lobe involvement. It may also relate to the early naming deficits identified in presymptomatic MAPT carriers.^43^ Complementing these structural findings, recent work has shown that MAPT mutation carriers exhibit marked alterations in functional connectivity at the earliest stages, possibly even preceding detectable structural changes, suggesting that network-level disruptions may be among the first indicators of disease onset (Bouzigues *et al*, 2025).^44^

Our study leveraged fine-grained clinical characterisation and larger sample sizes to disentangle mutation carriers across disease stages. This enabled the identification of subtle brain changes throughout clinical progression, advancing our understanding beyond previous studies.

### Cortical thickness and volume: different but complementary for genetic neurodegenerative disease

Most studies in (genetic) FTD have focused on grey matter volume, with relatively few investigating cortical thickness, and even fewer investigating both metrics simultaneously.^5^ These measures have distinct genetic determinants^45^ and ontogenetic pathways.^46^ While volume is considered more reliable, it is more influenced by head size, whereas cortical thickness is affected by gyrification patterns.^47 48^ Such differences likely explain why divergent atrophy patterns have been identified depending on the metric used in neurodegenerative diseases^47 48^ or more specifically (but also rarely studied) in FTD.^5^

Our findings support these distinctions, revealing striking differences in atrophy patterns between the two metrics despite significant overlap. For instance, in asymptomatic C9orf72 expansion carriers, cortical thinning was far more widespread than volume reductions. Conversely, GRN mutation carriers exhibited the opposite pattern, with more prominent volume changes compared with cortical thinning at the prodromal stage. In MAPT mutation carriers, both volume and thickness changes were present in the left anterior temporal lobe, but only volume reductions were observed on the right during the prodromal stage. The contrasting patterns in presymptomatic C9orf72 and GRN carriers are novel findings not previously emphasised.^5^ For MAPT carriers, prior work has suggested left-right asymmetries between the two metrics, though this was observed in reverse at follow-up, with cortical thinning becoming more prominent on the right.^5^ The limited sample size of previous studies underscores the need for further validation of our findings regarding asymmetric involvement of these measures in presymptomatic MAPT carriers.

With symptom onset and progression, we observed greater convergence between the two metrics in all groups. Cortical thinning appeared more widespread across the cortex, including posterior regions, whereas volume changes showed earlier, more localised changes in characteristic areas of neurodegeneration in FTD, such as orbitofrontal lobe and frontal gyri (bilaterally in C9orf72 and left-sided in GRN), and anterior temporal lobes (MAPT). These findings highlight the complementary roles of cortical thickness and volume measures for a comprehensive evaluation of pathological processes across genetic subtypes of FTD.

### Towards the definition of an imaging composite score in genetic FTD for future clinical trials

We highlight the earliest and most reliable grey matter changes and suggest these as a grey matter composite score for each genetic group. For C9orf72 expansion carriers, we put forward thalami volume, postcentral gyrus thickness and precuneus thickness. The first two regions have been highlighted in other samples.^4 5 7 39^ Specific reduced postcentral gyrus thickness in presymptomatic C9orf72 carriers was reported in a previous study.^5^ Interestingly, this is mostly associated with ALS or nfvPPA syndromes, rather than the more common bvFTD phenotype. It may relate to the more somatic delusions which are often reported in C9orf72 carriers.^49^ The precuneus has not often been mentioned as an early region of change in presymptomatic C9orf72 expansion carriers, yet it showed high effect sizes in both our asymptomatic and prodromal groups. A study which showed faster cortical thinning in the motor cortex and in the parietal regions, including the precuneus, in C9orf72 asymptomatic carriers supports our results.^30^ This is also consistent with our ‘quasi-longitudinal’ estimated progression which shows the largest decline for bilateral precuneus. Moreover, atrophy of the precuneus may relate to the more common memory impairments observed in C9orf72 carriers.^50^

For GRN mutation carriers, typical left frontal pole and pars orbitalis volume as well as left caudal middle frontal thickness were identified as the earliest changes. These are perfectly in line with the regions of atrophy typically associated with bvFTD and PPA syndromes, highlighting that grey matter changes are likely to be occurring prior to symptom onset, even in this group which has typically been suggested to show changes only from symptom onset. Thickness of the caudal middle frontal gyrus (Brodmann areas 6 and 8) appeared as the region with the sharpest decline between prodromal and symptom onset.

Finally, in MAPT mutation carriers, we highlight right temporal pole volume and left insula thickness as possible regions of early change which showed equal patterns of decline in the estimated progression models.

For each genetic group, these combinations of regions and metrics showed good to very good ability to dissociate asymptomatic and prodromal carriers from controls. Though these will have to be further validated in larger samples and in real longitudinal datasets, we put forward these grey matter composites for each genetic FTD group. Standardising these would be useful not only in future therapeutic trials, but also to enhance clinicians’ diagnostic process, thus overcoming the limitations of cognitive assessments at initial disease stages.

### Processes of neurodevelopment or early neurodegeneration?

Our findings, alongside prior work, invite speculation regarding whether presymptomatic grey matter changes in genetic FTD stem from neurodevelopmental or early neurodegenerative processes. Pathogenetic mutations, even if causal of late-onset disorders, likely influence neurodevelopment. For example, the youngest carriers of FTD-causing mutations exhibit grey matter differences compared with controls before their thirties,^15^ with smaller total brain and thalamic volumes and altered white matter tracts.^8 15^ Moreover, minimal increases in volume loss rate over time in C9orf72 carriers, unlike MAPT and GRN carriers, pointed to neurodevelopmental origins rather than progressive degeneration.^5 6^ While extremely early, slow-progressing neurodegenerative pathology cannot be excluded,^3^ these findings allude to neurodevelopmental vulnerabilities^13 26^ further supported by non-evolving cognitive deficits and psychiatric symptoms years before typical FTD emergence.^8^,^1151^

The divergence in the nature and timing of grey matter changes between C9orf72 carriers and GRN or MAPT carriers raises interesting questions. Unlike C9orf72, GRN and MAPT mutations appear to show grey matter reductions more closely with symptom onset, with these groups even showing larger brain volumes earlier in life, possibly conferring some protective features.^15^ The distinct patterns of cortical thinning in C9orf72 carriers versus early volume reductions in GRN carriers support the hypothesis that cortical thickness changes may better reflect neurodevelopmental processes, whereas volume reductions indicate neurodegenerative burden. This idea is consistent with our findings: cortical thickness measures were more prominent in C9orf72 ROIs, while volume measures were emphasised in GRN ROIs. Additionally, early grey matter changes in GRN correlated with clinical presentation, a relationship not observed in C9orf72 or MAPT carriers, though samples remained very limited for these analyses. This has also been hinted at in previous work showing faster cortical thinning starting early in adulthood in C9orf72 carriers, followed by a steady pace of evolution rather than an acceleration around the typical age of symptom onset.^27^ However, evidence of smaller brain and thalamic volumes but unaltered cortical thickness in young C9orf72 carriers^15^ adds complexity, emphasising the need for further investigation.

Our results highlight the complementary value of combining cortical thickness and volume measures to comprehensively assess pathological processes in different genetic groups. If neurodevelopmental theories gain further support, longitudinal studies, particularly in C9orf72 carriers with ALS phenotypes, will be crucial, as early diagnosis could have significant therapeutic implications.^54^

### Strengths and limitations of the study

Our study has the advantage of involving both volume and cortical thickness measurements which have been mostly investigated in isolation, causing inconsistency of results and obscuring the differential utility of each of these measures. Moreover, our bottom-up data-driven approach starting at the vertex-wise level before summarising specific brain regions of interest using a parcellation scheme gives us an unbiased perspective. Finally, our large sample sizes allowed us to subdivide carriers more precisely than previous studies, while still avoiding underpowered groups and preventing the grouping of carriers with diverse clinical characteristics.

Unfortunately, we were not able to further divide mutation carriers according to specific mutations. Moreover, the analyses investigating correlations between grey matter and follow-up clinical severity were computed in very small samples, which therefore limits the reliability of those specific results. Overall, it is important to note that this quasi-longitudinal design does not replace a real longitudinal study which is very much needed to validate our results and further contribute to identifying the best grey matter markers of progression. This quasi-longitudinal study was the necessary step in preparation for a real longitudinal exploration of the fine-grained grey matter changes in genetic FTD. Furthermore, though our univariate study found relevant and interesting results, a multivariate analysis assessing the variation of grey matter metrics in different areas simultaneously would also greatly benefit the field, enabling stronger statistical power and the exploration of several markers alongside each other. Finally, we would like to highlight that this multicentric study included a solely white ethnic population and thus our findings may not be generalisable to wider ethnic groups for whom such analyses will need further investigation.

## Conclusions and future perspectives

Our findings highlight the complementary value of cortical thickness and grey matter volume as distinct yet converging indicators of neurodegeneration. Whereas thickness alterations appear more pronounced in posterior regions (eg, in C9orf72 cases), volume loss tends to dominate anterior areas (eg, in GRN mutation carriers). This spatial dissociation highlights the non-redundant nature of these metrics, which, when integrated, may offer a richer and more nuanced picture of early structural disruption. Moving forward, combining these morphometric markers with white matter and functional connectivity data could pave the way towards a multidimensional and temporally sensitive signature of disease progression.

Future work will require the validation of these suggested composites in larger samples, particularly within longitudinal studies, and will benefit from the development of more refined techniques to investigate neurodevelopmental trajectories in these individuals, possibly from the first decades of life.

## Supplementary material

10.1136/jnnp-2025-337186 online supplemental file 1

## Data Availability

Data are available on reasonable request.

## References

[1] Rohrer JD, Warren JD (2011). Phenotypic signatures of genetic frontotemporal dementia. Curr Opin Neurol.

[2] Staffaroni AM, Quintana M, Wendelberger B (2022). Temporal order of clinical and biomarker changes in familial frontotemporal dementia. Nat Med.

[3] Rohrer JD, Nicholas JM, Cash DM (2015). Presymptomatic cognitive and neuroanatomical changes in genetic frontotemporal dementia in the Genetic Frontotemporal dementia Initiative (GENFI) study: a cross-sectional analysis. Lancet Neurol.

[4] Cash DM, Bocchetta M, Thomas DL (2018). Patterns of gray matter atrophy in genetic frontotemporal dementia: results from the GENFI study. Neurobiol Aging.

[5] Panman JL, Jiskoot LC, Bouts M (2019). Gray and white matter changes in presymptomatic genetic frontotemporal dementia: a longitudinal MRI study. Neurobiol Aging.

[6] Staffaroni AM, Goh SYM, Cobigo Y (2020). Rates of Brain Atrophy Across Disease Stages in Familial Frontotemporal Dementia Associated With MAPT, GRN, and C9orf72 Pathogenic Variants. JAMA Netw Open.

[7] Bocchetta M, Todd EG, Bouzigues A (2023). Structural MRI predicts clinical progression in presymptomatic genetic frontotemporal dementia: findings from the GENetic Frontotemporal dementia Initiative cohort. *Brain Commun*.

[8] Bertrand A, Wen J, Rinaldi D (2018). Early Cognitive, Structural, and Microstructural Changes in Presymptomatic C9orf72 Carriers Younger Than 40 Years. JAMA Neurol.

[9] Montembeault M, Sayah S, Rinaldi D (2020). Cognitive inhibition impairments in presymptomatic C9orf72 carriers. J Neurol Neurosurg Psychiatry.

[10] Lulé DE, Müller H-P, Finsel J (2020). Deficits in verbal fluency in presymptomatic *C9orf72* mutation gene carriers-a developmental disorder. *J Neurol Neurosurg Psychiatry*.

[11] Gossink F, Dols A, Stek ML (2022). Early life involvement in C9orf72 repeat expansion carriers. J Neurol Neurosurg Psychiatry.

[12] Bruffaerts R, Gors D, Bárcenas Gallardo A (2022). Hierarchical spectral clustering reveals brain size and shape changes in asymptomatic carriers of C9orf72. Brain Commun.

[13] Lee SE, Sias AC, Mandelli ML (2017). Network degeneration and dysfunction in presymptomatic *C9ORF72* expansion carriers. Neuroimage Clin.

[14] Shoukry RS, Waugh R, Bartlett D (2020). Longitudinal changes in resting state networks in early presymptomatic carriers of C9orf72 expansions. NeuroImage Clin.

[15] Finger E, Malik R, Bocchetta M (2023). Neurodevelopmental effects of genetic frontotemporal dementia in young adult mutation carriers. Brain (Bacau).

[16] Hutton C, Draganski B, Ashburner J (2009). A comparison between voxel-based cortical thickness and voxel-based morphometry in normal aging. Neuroimage.

[17] de Chastelaine M, Srokova S, Hou M (2023). Cortical thickness, gray matter volume, and cognitive performance: a crosssectional study of the moderating effects of age on their interrelationships. Cereb Cortex.

[18] Winkler AM, Kochunov P, Blangero J (2010). Cortical thickness or grey matter volume? The importance of selecting the phenotype for imaging genetics studies. Neuroimage.

[19] Worker A, Blain C, Jarosz J (2014). Cortical thickness, surface area and volume measures in Parkinson’s disease, multiple system atrophy and progressive supranuclear palsy. PLoS One.

[20] Gerrits NJHM, van Loenhoud AC, van den Berg SF (2016). Cortical Thickness, Surface Area and Subcortical Volume Differentially Contribute to Cognitive Heterogeneity in Parkinson’s Disease. PLoS One.

[21] Miyagawa T, Brushaber D, Syrjanen J (2020). Utility of the global CDR plus NACC FTLD rating and development of scoring rules: Data from the ARTFL/LEFFTDS Consortium. Alzheimers Dement.

[22] Goto M, Abe O, Hagiwara A (2022). Advantages of Using Both Voxel- and Surface-based Morphometry in Cortical Morphology Analysis: A Review of Various Applications. Magn Reson Med Sci.

[23] Fischl B (2012). FreeSurfer. Neuroimage.

[24] Routier A, Burgos N, Díaz M (2021). Clinica: An Open-Source Software Platform for Reproducible Clinical Neuroscience Studies. Front Neuroinform.

[25] Desikan RS, Ségonne F, Fischl B (2006). An automated labeling system for subdividing the human cerebral cortex on MRI scans into gyral based regions of interest. Neuroimage.

[26] Walhout R, Schmidt R, Westeneng H-J (2015). Brain morphologic changes in asymptomatic C9orf72 repeat expansion carriers. Neurology (ECronicon).

[27] Le Blanc G, Jetté Pomerleau V, McCarthy J (2020). Faster Cortical Thinning and Surface Area Loss in Presymptomatic and Symptomatic C9orf72 Repeat Expansion Adult Carriers. Ann Neurol.

[28] Moore KM, Nicholas J, Grossman M (2020). Age at symptom onset and death and disease duration in genetic frontotemporal dementia: an international retrospective cohort study. Lancet Neurol.

[29] Floeter MK, Bageac D, Danielian LE (2016). Longitudinal imaging in *C9orf72* mutation carriers: Relationship to phenotype. Neuroimage Clin.

[30] van Veenhuijzen K, Tan HHG, Nitert AD (2025). Longitudinal Magnetic Resonance Imaging in Asymptomatic C9orf72 Mutation Carriers Distinguishes Phenoconverters to Amyotrophic Lateral Sclerosis or Amyotrophic Lateral Sclerosis With Frontotemporal Dementia. Ann Neurol.

[31] Dopper EGP, Rombouts SARB, Jiskoot LC (2014). Structural and functional brain connectivity in presymptomatic familial frontotemporal dementia. Neurology (ECronicon).

[32] Caroppo P, Habert MO, Durrleman S (2015). Lateral Temporal Lobe: An Early Imaging Marker of the Presymptomatic GRN Disease. J Alzheimers Dis.

[33] Popuri K, Dowds E, Beg MF (2018). Gray matter changes in asymptomatic C9orf72 and GRN mutation carriers. NeuroImage: Clinical.

[34] Borrego-Écija S, Sala-Llonch R, van Swieten J (2021). Disease-related cortical thinning in presymptomatic granulin mutation carriers. NeuroImage: Clinical.

[35] Rohrer JD, Warren JD, Barnes J (2008). Mapping the progression of progranulin-associated frontotemporal lobar degeneration. Nat Rev Neurol.

[36] Jiskoot LC, Bocchetta M, Nicholas JM (2018). Presymptomatic white matter integrity loss in familial frontotemporal dementia in the GENFI cohort: A cross-sectional diffusion tensor imaging study. Ann Clin Transl Neurol.

[37] Saracino D, Dorgham K, Camuzat A (2021). Plasma NfL levels and longitudinal change rates in *C9orf72* and *GRN*-associated diseases: from tailored references to clinical applications. J Neurol Neurosurg Psychiatry.

[38] Rohrer JD, Ridgway GR, Modat M (2010). Distinct profiles of brain atrophy in frontotemporal lobar degeneration caused by progranulin and tau mutations. Neuroimage.

[39] Whitwell JL, Weigand SD, Boeve BF (2012). Neuroimaging signatures of frontotemporal dementia genetics: C9ORF72, tau, progranulin and sporadics. Brain (Bacau).

[40] Gordon E, Rohrer JD, Fox NC (2016). Advances in neuroimaging in frontotemporal dementia. J Neurochem.

[41] Borrego-Ecija S, Juncà-Parella J, Vandebergh M (2024). Association of Initial Side of Brain Atrophy With Clinical Features and Disease Progression in Patients With *GRN* Frontotemporal Dementia. Neurology (ECronicon).

[42] Domínguez-Vivero C, Wu L, Lee S (2020). Structural Brain Changes in Pre-Clinical FTD MAPT Mutation Carriers. J Alzheimers Dis.

[43] Bouzigues A, Russell LL, Peakman G (2022). Anomia is present pre-symptomatically in frontotemporal dementia due to MAPT mutations. J Neurol.

[44] Bouzigues A, Du VL, Joulot M (2025). Structural and functional connectivity in tau mutation carriers: from presymptomatic to symptomatic frontotemporal dementia. Alzheimer’s &Amp; Dementia.

[45] Panizzon MS, Fennema-Notestine C, Eyler LT (2009). Distinct genetic influences on cortical surface area and cortical thickness. Cereb Cortex.

[46] Chenn A, Walsh CA (2003). Increased neuronal production, enlarged forebrains and cytoarchitectural distortions in beta-catenin overexpressing transgenic mice. Cereb Cortex.

[47] Schwarz CG, Gunter JL, Wiste HJ (2016). A large-scale comparison of cortical thickness and volume methods for measuring Alzheimer’s disease severity. Neuroimage Clin.

[48] Choi M, Youn H, Kim D (2019). Comparison of neurodegenerative types using different brain MRI analysis metrics in older adults with normal cognition, mild cognitive impairment, and Alzheimer’s dementia. PLoS One.

[49] Snowden JS, Rollinson S, Thompson JC (2012). Distinct clinical and pathological characteristics of frontotemporal dementia associated with C9ORF72 mutations. Brain (Bacau).

[50] van der Ende EL, Bron EE, Poos JM (2022). A data-driven disease progression model of fluid biomarkers in genetic frontotemporal dementia. Brain (Bacau).

[51] Convery RS, Bocchetta M, Greaves CV (2020). Abnormal pain perception is associated with thalamo-cortico-striatal atrophy in *C9orf72* expansion carriers in the GENFI cohort. *J Neurol Neurosurg Psychiatry*.

[52] Russell LL, Greaves CV, Bocchetta M (2020). Social cognition impairment in genetic frontotemporal dementia within the GENFI cohort. Cortex.

[53] Poos JM, Russell LL, Peakman G (2021). Impairment of episodic memory in genetic frontotemporal dementia: A GENFI study. *Alz & Dem Diag Ass & Dis Mo*.

[54] Bede P, Siah WF, McKenna MC (2020). Consideration of C9orf72-associated ALS-FTD as a neurodevel-opmental disorder: insights from neuroimaging. J Neurol Neurosurg Psychiatry.

