# Supplementary material for: Composite grey matter fingerprints for genetic frontotemporal dementia
Source: J Neurol Neurosurg Psychiatry. 2026 Feb 12;97(7):e337186. doi: 10.1136/jnnp-2025-337186 (PMC13288956; doi:10.1136/jnnp-2025-337186)
Supplement: online supplemental file 1 [file jnnp-97-7-s001.pdf]

Supplementary materials

Supplementary Table 1: Protocol details for each scanner from the different acquisition sites, within both phases of the GENFI protocol. Protocols were developed to match each other as best as technically possible.

	Scanner manufacturer	Scanner model	Software	Duration	Voxel/matrix size	Key parameters
**GENFI protocol phase 2**	**Siemens**	**Prisma**	VE11C	5min 6s	1.1x1.1x1.1mm3 256x256x208	MPRAGE sagittal, 10% phase oversampling, iPAT=2, TI/TR=850/2000ms; flip angle 8°
		**Skyra**	VE11C	8min 32s	1.1x1.1x1.1mm3 256x256x208	MPRAGE sagittal, iPAT=off, TI/TR=850/2000ms; flip angle 8°
		**Trio**	VB17/19	5min 6s	1.1x1.1x1.1mm3 256x256x208	MPRAGE sagittal, 10% phase oversampling, iPAT=2, TI/TR=850/2000ms; flip angle 8°
	**Philips**	**Achieva**	R5.1.7.2	4min 43s	1.1x1.1x1.1mm3 256x256x208	MPRAGE sagittal, SENSE=2, TI/TR=933/2200ms; flip angle 8°
		**Ingenia**	R5.1.7.2	4min 43s	1.1x1.1x1.1mm3 256x256x208	MPRAGE sagittal, SENSE=2, TI/TR=934/2200ms; flip angle 8°
	**GE**	**Discovery 750**	DV24	5min 6s	1.1x1.1x1.1mm3 256x256x208	IR-SPGR sagittal, ASSET=2, TI=400ms; inner loop TR=6.6ms, flip angle 8°
**GENFI protocol phase 1**	**Siemens**	**Skyra**	VD13	9min 23s	1.1x1.1x1.1mm3 256x256x208	MPRAGE sagittal, flip angle 10°, TI/TR=900/2200ms;
		**Trio**	VB17	9min 23s	1.1x1.1x1.1mm3 256x256x208	MPRAGE sagittal, flip angle 10°, TI/TR=900/2200ms;
		**Allegra**	2004A	*9min 23s*	*1.1x1.1x1.1mm3 256x256x208*	*MPRAGE sagittal, flip angle 10°, TI/TR=900/2200ms;*
	**Philips**	**Achieva**		9min 25s	1.1x1.1x1.1mm3 256x256x208	MPRAGE sagittal, flip angle 10°, TI/TR=960/2200ms;
	**GE**	**Discovery 750**			1.02x1.02x1.2mm3 256x256x200	Sag IR-SPGR, TI=400ms, flip angle 11°

Supplementary Table 2: Significant differences of demographics characteristics between each gene_stage group and controls, and between different genetic groups of the same severity stage

	Controls	FTLD-CDR				
		0	0.5	1	2	3
**Age**	All excluding:<br>C9orf72_0/05<br>GRN_0<br>MAPT_05/1	MAPT vs GRN<br>p<0.001	MAPT vs GRN<br>p=0.032		MAPT vs GRN<br>p=0.007	MAPT vs C9orf72<br>p=0.024
**Sex**	C9orf72_1/2					
**Education**	C9orf72_3<br>GRN_2/3<br>MAPT_2					MAPT vs C9orf72<br>MAPT vs GRN<br>p<0.001

Supplementary Table 3: Summary of statistical results for cortical thickness differences in each gene_stage group compared to controls.

clusid	nverts	resels	P
**C9_0**			
1	107602	78,3	<0.0001
2	121416	74,6	<0.0001
**C9_0.5**			
1	108293	122,9	<0.0001
2	100895	61,3	<0.0001
3	18	3,3	0.003
4	3655	3,1	0.006
5	134	2,8	0.01
6	39	2,8	0.1
**C9_1**			
1	67203	47,1	<0.0001
2	31994	18,6	<0.0001
3	8449	12,3	<0.0001
4	236	8,2	<0.0001
5	2249	4,4	0.0002
6	34	4,3	0.0003
7	73	4,2	0.0004
8	7160	2,5	0.02
**C9_2**			
1	135078	146,2	<0.0001
2	136187	131,5	<0.0001
3	26	2,8	0.01
**C9_3**			
1	139906	222,2	<0.0001
2	139702	203,3	<0.0001
3	59	6,7	<0.0001

clusid	nverts	resels	P
**GRN_0**			
1	18	3,3	0,003
**GRN_0.5**			
1	107	10,9	<0.0001
2	1213	7,1	<0.0001
3	13826	6,9	<0.0001
4	6152	4,2	0.0003
**GRN_1**			
1	97045	64,6	<0.0001
2	34612	16,9	<0.0001
**GRN_2**			
1	91923	101,2	<0.0001
2	60338	74,7	<0.0001
3	110	6,3	<0.0001
4	36	4,9	<0.0001
5	6486	4,3	0.0003
6	31	3,3	0.003
7	26	2,6	0.02
**GRN_3**			
1	138951	206,9	<0.0001
2	137404	202,8	<0.0001

clusid	nverts	resels	P
**MAPT_0**			
1	1941	7,4	<0.0001
2	1543	5,8	<0.0001
3	49	5,7	<0.0001
4	59	3,3	0.003
**MAPT_0.5**			
1	10591	13,4	<0.0001
2	282	10,9	<0.0001
3	181	9,7	<0.0001
4	2873	3,3	0.003
5	39	3,0	0.007
6	3179	2,3	0.04
**MAPT_1**			
1	18050	35,2	<0.0001
2	11133	28,4	<0.0001
3	7795	3,5	0.002
4	92	2,8	0.01
**MAPT_2**			
1	79216	100,7	<0.0001
2	76586	90,6	<0.0001
3	875	14,7	<0.0001
**MAPT_3**			
1	28375	70,4	<0.0001
2	25503	55,6	<0.0001
3	58	5,7	<0.0001
4	9773	4,6	0.0001
5	33	3,4	0.003
6	4645	3,2	0.004
7	4878	3,0	0.006
8	19	2,5	0.02
9	5494	2,2	0.05

Supplementary Table 4: Summary of statistical results for cortical volume differences in each gene_stage group compared to controls.

clusid	nverts	resels	P
**C9_0**			
1	87137	58,3	<0.0001
2	99558	56,1	<0.0001
**C9_0.5**			
1	78008	89,7	<0.0001
2	74767	32,8	<0.0001
3	8322	18,9	<0.0001
4	365	4,7	<0.0001
5	87	3,1	0.004
6	19	2,9	0.006
**C9_1**			
1	39368	16,9	<0.0001
2	34621	15,6	<0.0001
3	411	6,5	<0.0001
4	1487	5,6	<0.0001
5	2913	4,7	<0.0001
6	65	2,5	0.02
**C9_2**			
1	138705	144,8	<0.0001
2	137075	131,0	<0.0001
**C9_3**			
1	143034	176,1	<0.0001
2	138960	168,6	<0.0001

clusid	nverts	resels	P
**GRN_0**			
**GRN_0.5**			
1	16425	7,3	<0.0001
2	103	6,2	<0.0001
3	9392	4,5	0.0001
4	7217	2,9	0.006
5	206	2,8	0.007
**GRN_1**			
1	106908	54,7	<0.0001
2	34118	17,4	<0.0001
3	4454	3,0	0.005
**GRN_2**			
1	83639	79,1	<0.0001
2	7725	43,5	<0.0001
3	36006	15,3	<0.0001
4	9212	8,6	<0.0001
5	183	7,3	<0.0001
**GRN_3**			
1	135122	139,1	<0.0001
2	123435	122,5	<0.0001
3	248	2,6	0.01
4	32	2,3	0.03

clusid	nverts	resels	P
**MAPT_0**			
1	847	4,8	<0.0001
**MAPT_0.5**			
1	6083	9,3	<0.0001
2	4566	7,8	<0.0001
3	20	2,7	0.01
4	64	2,5	0.02
5	5387	2,2	0.04
**MAPT_1**			
1	21816	35,6	<0.0001
2	15714	24,2	<0.0001
3	10456	11,6	<0.0001
4	28	3,1	0.004
**MAPT_2**			
1	51303	72,5	<0.0001
2	49100	72,0	<0.0001
3	859	11,9	<0.0001
4	25	2,3	0.03
**MAPT_3**			
1	42433	68,2	<0.0001
2	29234	49,9	<0.0001
3	7898	11,2	<0.0001
4	139	5,2	<0.0001
5	143	5,1	<0.0001
6	43	3,1	0.004

Supplementary Table 5: Summary of statistical results for subcortical volume differences in each gene_stage group compared to controls.

Group	Hemisphere	Structure	Standard Error	t-ratio	p-value	Adjusted p-value
C9orf72 0	Left	Accumbens	12.18	2.74	>0.05	>0.05
**C9orf72 0**		**Amygdala**	**24.72**	**3.85**	**0.01**	**0.053**
C9orf72 0		Caudate	49.06	1.15	>0.05	>0.05
C9orf72 0		Cerebellum	550.85	3.13	>0.05	>0.05
**C9orf72 0**		**Hippocampus**	**42.98**	**4.77**	**0.0002**	**0.002**
C9orf72 0		Pallidum	29.98	0.85	>0.05	>0.05
**C9orf72 0**		**Thalamus**	**92.00**	**7.90**	**<0.0001**	**3.50e-11**
C9orf72 0		VentralDC	51.84	0.92	>0.05	>0.05
C9orf72 0	Right	Accumbens	10.71	2.79	>0.05	>0.05
C9orf72 0		Amygdala	24.51	3.23	>0.05	>0.05
C9orf72 0		Caudate	50.90	2.00	>0.05	>0.05
C9orf72 0		Cerebellum	563.82	2.70	>0.05	>0.05
**C9orf72 0**		**Hippocampus**	**46.27**	**3.91**	**0.01**	**0.043**
C9orf72 0		Pallidum	29.91	1.15	>0.05	>0.05
**C9orf72 0**		**Thalamus**	**79.98**	**7.49**	**<0.0001**	**4.41e-10**
C9orf72 0		VentralDC	49.33	0.78	>0.05	>0.05
C9orf72 0.5	Left	Accumbens	18.43	1.13	>0.05	>0.05
C9orf72 0.5		Amygdala	37.39	1.91	>0.05	>0.05
C9orf72 0.5		Caudate	74.20	1.50	>0.05	>0.05
C9orf72 0.5		Cerebellum	833.25	1.64	>0.05	>0.05
**C9orf72 0.5**		**Hippocampus**	**65.01**	**3.45**	**0.048**	>0.05
C9orf72 0.5		Pallidum	45.34	1.00	>0.05	>0.05
**C9orf72 0.5**		**Thalamus**	**139.16**	**5.37**	**<0.0001**	**9.86e-05**
C9orf72 0.5		VentralDC	78.41	1.40	>0.05	>0.05
C9orf72 0.5	Right	Accumbens	16.20	1.54	>0.05	>0.05
C9orf72 0.5		Amygdala	37.07	1.48	>0.05	>0.05
C9orf72 0.5		Caudate	76.99	1.62	>0.05	>0.05
C9orf72 0.5		Cerebellum	852.87	1.99	>0.05	>0.05
C9orf72 0.5		Hippocampus	69.99	2.84	>0.05	>0.05
C9orf72 0.5		Pallidum	45.25	1.77	>0.05	>0.05
**C9orf72 0.5**		**Thalamus**	**120.98**	**6.34**	**<0.0001**	**5.55e-07**
C9orf72 0.5		VentralDC	74.61	0.65	>0.05	>0.05
C9orf72 1	Left	Accumbens	26.70	2.15	>0.05	>0.05
**C9orf72 1**		**Amygdala**	**54.17**	**4.11**	**0.005**	**0.022**
C9orf72 1		Caudate	107.51	2.36	>0.05	>0.05
C9orf72 1		Cerebellum	1207.25	1.50	>0.05	>0.05
**C9orf72 1**		**Hippocampus**	**94.20**	**4.07**	**0.005**	**0.024**

C9orf72 1		Pallidum	65.70	0.401	>0.05	>0.05
**C9orf72 1**		**Thalamus**	**201.62**	**3.79**	**0.01**	**0.064**
C9orf72 1		VentralDC	113.60	1.02	>0.05	>0.05
C9orf72 1	Right	Accumbens	23.47	2.32	>0.05	>0.05
C9orf72 1		Amygdala	53.71	2.94	>0.05	>0.05
C9orf72 1		Caudate	111.54	1.45	>0.05	>0.05
C9orf72 1		Cerebellum	1235.67	1.42	>0.05	>0.05
C9orf72 1		Hippocampus	101.41	3.37	>0.05	>0.05
C9orf72 1		Pallidum	65.56	0.14	>0.05	>0.05
C9orf72 1		Thalamus	175.28	2.76	>0.05	>0.05
C9orf72 1		VentralDC	108.10	1.10	>0.05	>0.05
**C9orf72 2**	Left	**Accumbens**	**22.77**	**5.18**	**<0.0001**	**0.0002**
**C9orf72 2**		**Amygdala**	**46.19**	**6.93**	**<0.0001**	**1.66e-08**
C9orf72 2		Caudate	91.67	2.79	>0.05	>0.05
C9orf72 2		Cerebellum	1029.41	2.53	>0.05	>0.05
**C9orf72 2**		**Hippocampus**	**80.32**	**10.38**	**<0.0001**	**4.26e-12**
C9orf72 2		Pallidum	56.02	1.26	>0.05	>0.05
**C9orf72 2**		**Thalamus**	**171.92**	**6.54**	**<0.0001**	**1.71e-07**
**C9orf72 2**		**VentralDC**	**96.87**	**5.31**	**<0.0001**	**0.0001**
**C9orf72 2**	Right	**Accumbens**	**20.01**	**7.17**	**<0.0001**	**3.62e-09**
**C9orf72 2**		**Amygdala**	**45.80**	**6.26**	**<0.0001**	**8.52e-07**
C9orf72 2		Caudate	95.11	3.09	>0.05	>0.05
C9orf72 2		Cerebellum	1053.64	2.37	>0.05	>0.05
**C9orf72 2**		**Hippocampus**	**86.47**	**10.37**	**<0.0001**	**4.26e-12**
C9orf72 2		Pallidum	55.90	2.36	>0.05	>0.05
**C9orf72 2**		**Thalamus**	**149.46**	**7.91**	**<0.0001**	**3.45e-11**
**C9orf72 2**		**VentralDC**	**92.18**	**5.06**	**<0.0001**	**0.0004**
**C9orf72 3**	Left	**Accumbens**	**23.38**	**6.41**	**<0.0001**	**3.56e-07**
**C9orf72 3**		**Amygdala**	**47.43**	**6.96**	**<0.0001**	**1.38e-08**
**C9orf72 3**		**Caudate**	**94.13**	**6.72**	**<0.0001**	**5.97e-08**
**C9orf72 3**		**Cerebellum**	**1057.01**	**3.92**	**0.009**	**0.041**
**C9orf72 3**		**Hippocampus**	**82.47**	**9.97**	**<0.0001**	**4.26e-12**
C9orf72 3		Pallidum	57.52	1.57	>0.05	>0.05
**C9orf72 3**		**Thalamus**	**176.53**	**8.52**	**<0.0001**	**4.30e-12**
**C9orf72 3**		**VentralDC**	**99.46**	**5.62**	**<0.0001**	**2.90e-05**
**C9orf72 3**	Right	**Accumbens**	**20.55**	**8.06**	**<0.0001**	**1.53e-11**
**C9orf72 3**		**Amygdala**	**47.03**	**6.36**	**<0.0001**	**4.83e-07**
**C9orf72 3**		**Caudate**	**97.66**	**5.22**	**<0.0001**	**0.0002**
**C9orf72 3**		**Cerebellum**	**1081.89**	**3.57**	**0.03**	**0.129**
**C9orf72 3**		**Hippocampus**	**88.79**	**8.92**	**<0.0001**	**4.26e-12**
C9orf72 3		Pallidum	57.40	0.42	>0.05	>0.05
**C9orf72 3**		**Thalamus**	**153.46**	**8.73**	**<0.0001**	**4.26e-12**

**C9orf72 3**		**VentralDC**	**94.65**	**5.31**	**<0.0001**	**0.0001**
GRN 0	Left	Accumbens	12.13	0.68	>0.05	>0.05
GRN 0		Amygdala	24.62	-0.29	>0.05	>0.05
GRN 0		Caudate	48.86	-1.65	>0.05	>0.05
GRN 0		Cerebellum	548.69	0.91	>0.05	>0.05
GRN 0		Hippocampus	42.81	-0.84	>0.05	>0.05
GRN 0		Pallidum	29.86	-1.29	>0.05	>0.05
GRN 0		Thalamus	91.64	0.19	>0.05	>0.05
GRN 0		VentralDC	51.63	1.13	>0.05	>0.05
GRN 0	Right	Accumbens	10.67	-0.13	>0.05	>0.05
GRN 0		Amygdala	24.41	-0.45	>0.05	>0.05
GRN 0		Caudate	50.70	-1.44	>0.05	>0.05
GRN 0		Cerebellum	561.61	0.47	>0.05	>0.05
GRN 0		Hippocampus	46.09	-0.93	>0.05	>0.05
GRN 0		Pallidum	29.80	-1.79	>0.05	>0.05
GRN 0		Thalamus	79.66	0.97	>0.05	>0.05
GRN 0		VentralDC	49.13	0.59	>0.05	>0.05
GRN 0.5	Left	Accumbens	21.79	0.66	>0.05	>0.05
GRN 0.5		Amygdala	44.20	0.16	>0.05	>0.05
GRN 0.5		Caudate	87.73	-0.86	>0.05	>0.05
GRN 0.5		Cerebellum	985.17	1.84	>0.05	>0.05
GRN 0.5		Hippocampus	76.87	1.81	>0.05	>0.05
GRN 0.5		Pallidum	53.61	-0.99	>0.05	>0.05
GRN 0.5		Thalamus	164.53	-0.48	>0.05	>0.05
GRN 0.5		VentralDC	92.70	0.94	>0.05	>0.05
GRN 0.5	Right	Accumbens	19.15	-0.02	>0.05	>0.05
GRN 0.5		Amygdala	43.83	0.56	>0.05	>0.05
GRN 0.5		Caudate	91.02	-1.26	>0.05	>0.05
GRN 0.5		Cerebellum	1008.36	1.88	>0.05	>0.05
GRN 0.5		Hippocampus	82.75	0.61	>0.05	>0.05
GRN 0.5		Pallidum	53.50	-0.05	>0.05	>0.05
GRN 0.5		Thalamus	143.03	-1.78	>0.05	>0.05
GRN 0.5		VentralDC	88.22	0.10	>0.05	>0.05
GRN 1	Left	Accumbens	25.74	2.44	>0.05	>0.05
GRN 1		Amygdala	52.22	1.31	>0.05	>0.05
**GRN 1**		**Caudate**	**103.64**	**5.04**	**<0.0001**	**0.0005**
GRN 1		Cerebellum	1163.83	1.10	>0.05	>0.05
**GRN 1**		**Hippocampus**	**90.81**	**4.89**	**0.0001**	**0.0009**
GRN 1		Pallidum	63.33	-0.10	>0.05	>0.05
**GRN 1**		**Thalamus**	**194.37**	**4.43**	**0.001**	**0.006**
**GRN 1**		**VentralDC**	**109.52**	**3.70**	**0.02**	**0.084**
GRN 1	Right	Accumbens	22.63	1.64	>0.05	>0.05

GRN 1		Amygdala	51.78	0.38	>0.05	>0.05
GRN 1		Caudate	107.53	2.50	>0.05	>0.05
GRN 1		Cerebellum	1191.23	0.96	>0.05	>0.05
GRN 1		Hippocampus	97.76	2.22	>0.05	>0.05
GRN 1		Pallidum	63.20	0.74	>0.05	>0.05
GRN 1		Thalamus	168.97	2.86	>0.05	>0.05
GRN 1		VentralDC	104.22	2.07	>0.05	>0.05
GRN 2	Left	Accumbens	36.73	1.53	>0.05	>0.05
GRN 2		Amygdala	74.53	1.46	>0.05	>0.05
GRN 2		Caudate	147.92	3.28	>0.05	>0.05
GRN 2		Cerebellum	1661.05	1.36	>0.05	>0.05
**GRN 2**		**Hippocampus**	**129.60**	**6.56**	**<0.0001**	**1.56e-07**
GRN 2		Pallidum	90.39	-0.61	>0.05	>0.05
**GRN 2**		**Thalamus**	**277.41**	**4.12**	**0.004**	**0.021**
**GRN 2**		**VentralDC**	**156.30**	**3.82**	**0.01**	**0.058**
GRN 2	Right	Accumbens	32.30	2.10	>0.05	>0.05
GRN 2		Amygdala	73.90	1.17	>0.05	>0.05
GRN 2		Caudate	153.47	2.22	>0.05	>0.05
GRN 2		Cerebellum	1700.15	1.14	>0.05	>0.05
**GRN 2**		**Hippocampus**	**139.53**	**5.62**	**<0.0001**	**2.90e-05**
GRN 2		Pallidum	90.20	-1.12	>0.05	>0.05
GRN 2		Thalamus	241.16	3.30	>0.05	>0.05
GRN 2		VentralDC	148.74	3.12	>0.05	>0.05
**GRN 3**	Left	**Accumbens**	**33.43**	**6.10**	**<0.0001**	**2.16e-06**
**GRN 3**		**Amygdala**	**67.83**	**4.49**	**0.0009**	**0.005**
**GRN 3**		**Caudate**	**134.62**	**6.15**	**<0.0001**	**1.61e-06**
GRN 3		Cerebellum	1511.61	2.87	>0.05	>0.05
**GRN 3**		**Hippocampus**	**117.94**	**7.14**	**<0.0001**	**4.24e-09**
GRN 3		Pallidum	82.26	-0.10	>0.05	>0.05
GRN 3		Thalamus	252.45	3.31	>0.05	>0.05
**GRN 3**		**VentralDC**	**142.24**	**5.21**	**<0.0001**	**0.0002**
**GRN 3**	Right	**Accumbens**	**29.39**	**8.00**	**<0.0001**	**2.01e-11**
**GRN 3**		**Amygdala**	**67.25**	**3.55**	**0.03**	**0.133**
**GRN 3**		**Caudate**	**139.66**	**7.55**	**<0.0001**	**2.94e-10**
GRN 3		Cerebellum	1547.19	0.99	>0.05	>0.05
**GRN 3**		**Hippocampus**	**126.97**	**5.42**	**<0.0001**	**7.81e-05**
GRN 3		Pallidum	82.08	-2.23	>0.05	>0.05
**GRN 3**		**Thalamus**	**219.47**	**4.12**	**0.004**	**0.020**
**GRN 3**		**VentralDC**	**135.36**	**5.46**	**<0.0001**	**6.39e-05**
MAPT 0	Left	Accumbens	18.01	1.68	>0.05	>0.05
MAPT 0		Amygdala	36.54	0.77	>0.05	>0.05
MAPT 0		Caudate	72.52	-0.97	>0.05	>0.05

MAPT 0		Cerebellum	814.30	-1.78	>0.05	>0.05
MAPT 0		Hippocampus	63.54	1.28	>0.05	>0.05
MAPT 0		Pallidum	44.3197	-1.11	>0.05	>0.05
MAPT 0		Thalamus	136.00	-0.45	>0.05	>0.05
MAPT 0		VentralDC	76.63	-0.13	>0.05	>0.05
MAPT 0	Right	Accumbens	15.83	0.99	>0.05	>0.05
MAPT 0		Amygdala	36.23	1.17	>0.05	>0.05
MAPT 0		Caudate	75.24	-0.92	>0.05	>0.05
MAPT 0		Cerebellum	833.47	-2.07	>0.05	>0.05
MAPT 0		Hippocampus	68.40	1.19	>0.05	>0.05
MAPT 0		Pallidum	44.22	-0.79	>0.05	>0.05
MAPT 0		Thalamus	118.23	-0.67	>0.05	>0.05
MAPT 0		VentralDC	72.92	0.32	>0.05	>0.05
MAPT 0.5	Left	Accumbens	29.07	1.23	>0.05	>0.05
MAPT 0.5		Amygdala	58.98	1.60	>0.05	>0.05
MAPT 0.5		Caudate	117.06	-0.10	>0.05	>0.05
MAPT 0.5		Cerebellum	1314.52	0.19	>0.05	>0.05
MAPT 0.5		Hippocampus	102.56	3.34	>0.05	>0.05
MAPT 0.5		Pallidum	71.53	0.20	>0.05	>0.05
MAPT 0.5		Thalamus	219.54	1.27	>0.05	>0.05
MAPT 0.5		VentralDC	123.70	0.54	>0.05	>0.05
MAPT 0.5	Right	Accumbens	25.56	-1.11	>0.05	>0.05
MAPT 0.5		Amygdala	58.48	2.99	>0.05	>0.05
MAPT 0.5		Caudate	121.45	-0.18	>0.05	>0.05
MAPT 0.5		Cerebellum	1345.46	-0.03	>0.05	>0.05
MAPT 0.5		Hippocampus	110.42	3.07	>0.05	>0.05
MAPT 0.5		Pallidum	71.38	0.35	>0.05	>0.05
MAPT 0.5		Thalamus	190.85	0.44	>0.05	>0.05
MAPT 0.5		VentralDC	117.71	0.96	>0.05	>0.05
**MAPT 1**	Left	**Accumbens**	**37.60**	**3.92**	**0.009**	**0.041**
**MAPT 1**		**Amygdala**	**76.28**	**6.95**	**<0.0001**	**1.44e-08**
**MAPT 1**		**Caudate**	**151.40**	**3.58**	**0.03**	**0.124**
MAPT 1		Cerebellum	1700.04	1.84	>0.05	>0.05
**MAPT 1**		**Hippocampus**	**132.65**	**6.71**	**<0.0001**	**6.07e-08**
MAPT 1		Pallidum	92.51	1.42	>0.05	>0.05
MAPT 1		Thalamus	283.92	2.71	>0.05	>0.05
MAPT 1		VentralDC	159.97	2.53	>0.05	>0.05
**MAPT 1**	Right	**Accumbens**	**33.05**	**6.03**	**<0.0001**	**3.23e-06**
**MAPT 1**		**Amygdala**	**75.64**	**8.37**	**<0.0001**	**5.33e-12**
**MAPT 1**		**Caudate**	**157.07**	**3.62**	**0.03**	**0.109**
MAPT 1		Cerebellum	1740.06	1.71	>0.05	>0.05
**MAPT 1**		**Hippocampus**	**142.80**	**7.86**	**<0.0001**	**4.42e-11**

MAPT 1		Pallidum	92.32	2.25	>0.05	>0.05
MAPT 1		Thalamus	246.83	3.43	>0.05	>0.05
MAPT 1		VentralDC	152.23	3.22	>0.05	>0.05
**MAPT 2**	Left	**Accumbens**	**37.81**	**5.68**	**<0.0001**	**2.04e-05**
**MAPT 2**		**Amygdala**	**76.72**	**9.27**	**<0.0001**	**4.30e-12**
MAPT 2		Caudate	152.27	2.22	>0.05	>0.05
MAPT 2		Cerebellum	1709.92	-0.62	>0.05	>0.05
**MAPT 2**		**Hippocampus**	**133.42**	**9.57**	**<0.0001**	**4.26e-12**
MAPT 2		Pallidum	93.05	-1.20	>0.05	>0.05
**MAPT 2**		**Thalamus**	**285.57**	**4.39**	**0.001**	**0.007**
**MAPT 2**		**VentralDC**	**160.90**	**3.77**	**0.02**	**0.069**
**MAPT 2**	Right	**Accumbens**	**33.25**	**5.25**	**<0.0001**	**0.0002**
**MAPT 2**		**Amygdala**	**76.08**	**9.07**	**<0.0001**	**4.26e-12**
MAPT 2		Caudate	157.98	2.07	>0.05	>0.05
MAPT 2		Cerebellum	1750.17	-0.80	>0.05	>0.05
**MAPT 2**		**Hippocampus**	**143.63**	**9.42**	**<0.0001**	**4.26e-12**
MAPT 2		Pallidum	92.85	-1.25	>0.05	>0.05
**MAPT 2**		**Thalamus**	**248.26**	**4.59**	**0.0006**	**0.003**
**MAPT 2**		**VentralDC**	**153.12**	**3.97**	**0.008**	**0.035**
**MAPT 3**	Left	**Accumbens**	**39.78**	**6.89**	**<0.0001**	**2.14e-08**
**MAPT 3**		**Amygdala**	**80.71**	**10.00**	**<0.0001**	**4.26e-12**
MAPT 3		Caudate	160.19	2.15	>0.05	>0.05
MAPT 3		Cerebellum	1798.74	1.04	>0.05	>0.05
**MAPT 3**		**Hippocampus**	**140.35**	**12.15**	**<0.0001**	**<0.0001**
MAPT 3		Pallidum	97.88	-0.37	>0.05	>0.05
**MAPT 3**		**Thalamus**	**300.41**	**4.69**	**0.0004**	**0.002**
**MAPT 3**		**VentralDC**	**169.26**	**4.02**	**0.006**	**0.029**
**MAPT 3**	Right	**Accumbens**	**34.97**	**6.99**	**<0.0001**	**1.13e-08**
**MAPT 3**		**Amygdala**	**80.03**	**10.86**	**<0.0001**	**4.266e-12**
MAPT 3		Caudate	166.19	1.67	>0.05	>0.05
MAPT 3		Cerebellum	1841.08	0.73	>0.05	>0.05
**MAPT 3**		**Hippocampus**	**151.09**	**11.20**	**<0.0001**	**<0.0001**
MAPT 3		Pallidum	97.68	0.43	>0.05	>0.05
MAPT 3		Thalamus	261.15	3.15	>0.05	>0.05
**MAPT 3**		**VentralDC**	**161.07**	**3.77**	**0.02**	**0.068**

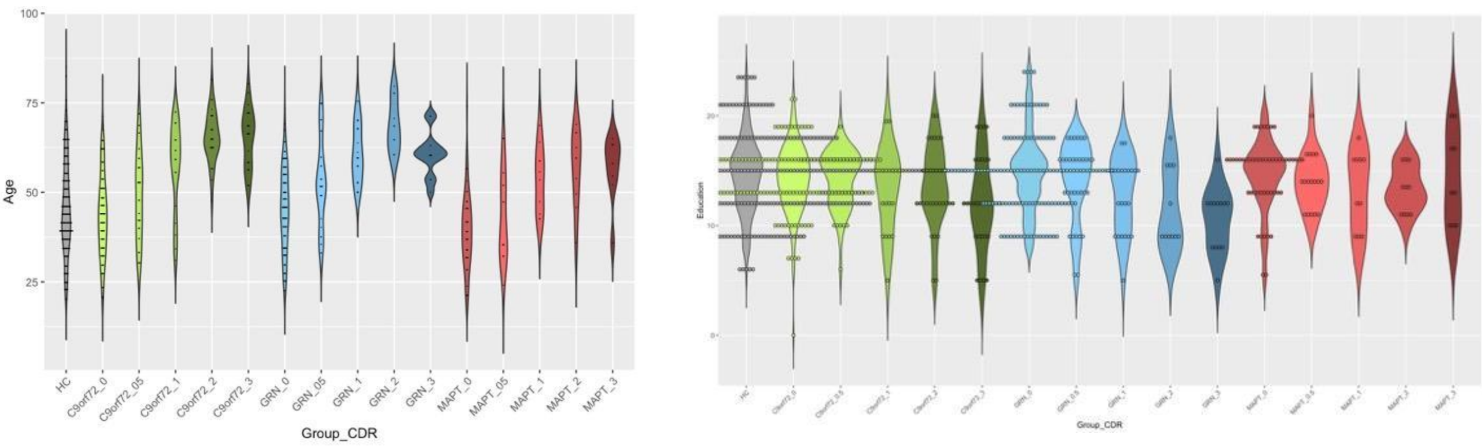

Supplementary Figure 1. Distribution of age and education in each gene_stage group.

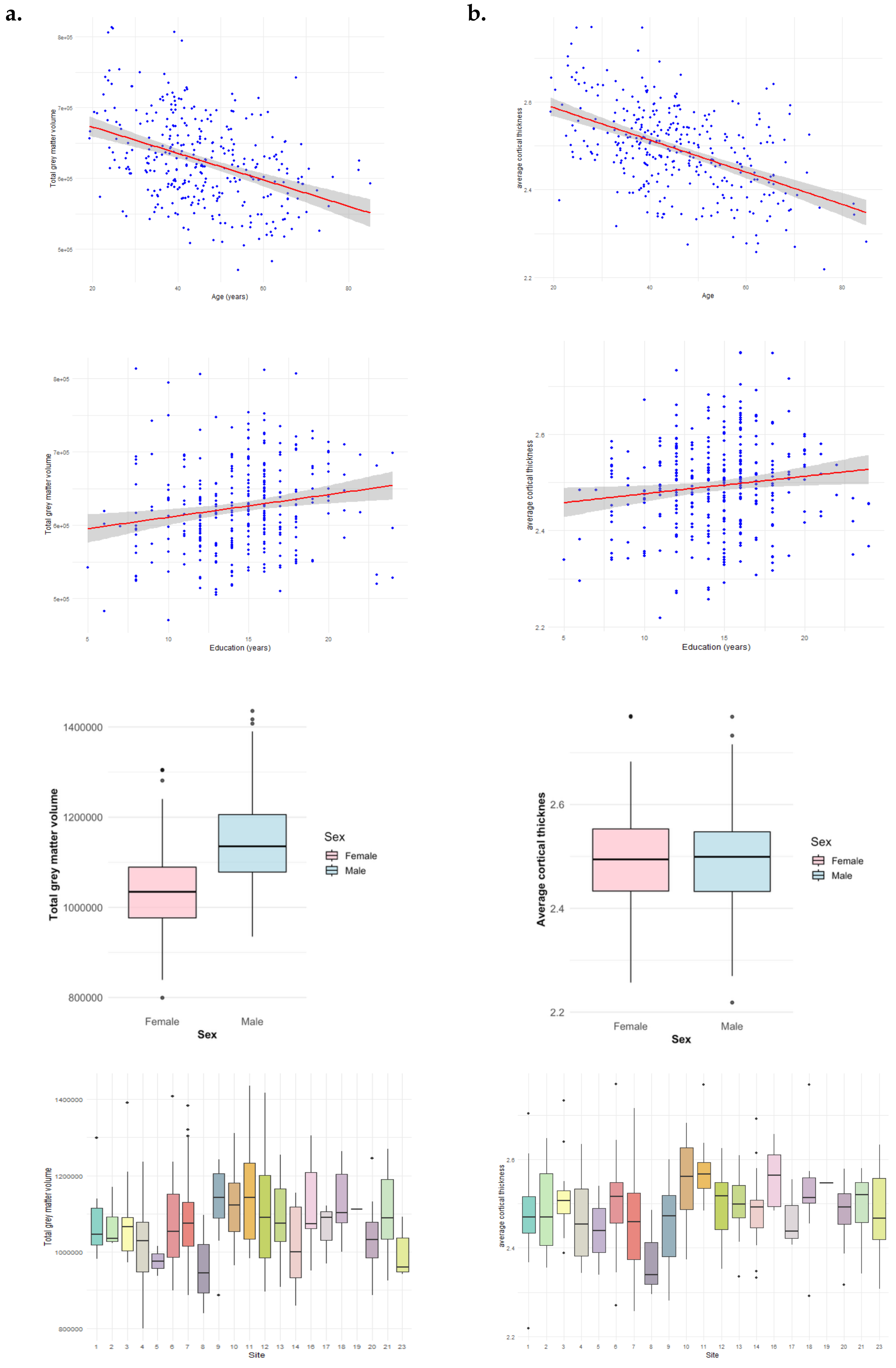
a.
b.
Total grey matter volume
Age (years)
average cortical thickness
Age
Education (years)
Sex
Female
Male
Average cortical thicknes
Site

Supplementary Figure 2: Relationship between total grey matter volume (panel a) and average cortical thickness (panel b) with age, education, sex and site of data acquisition in controls.

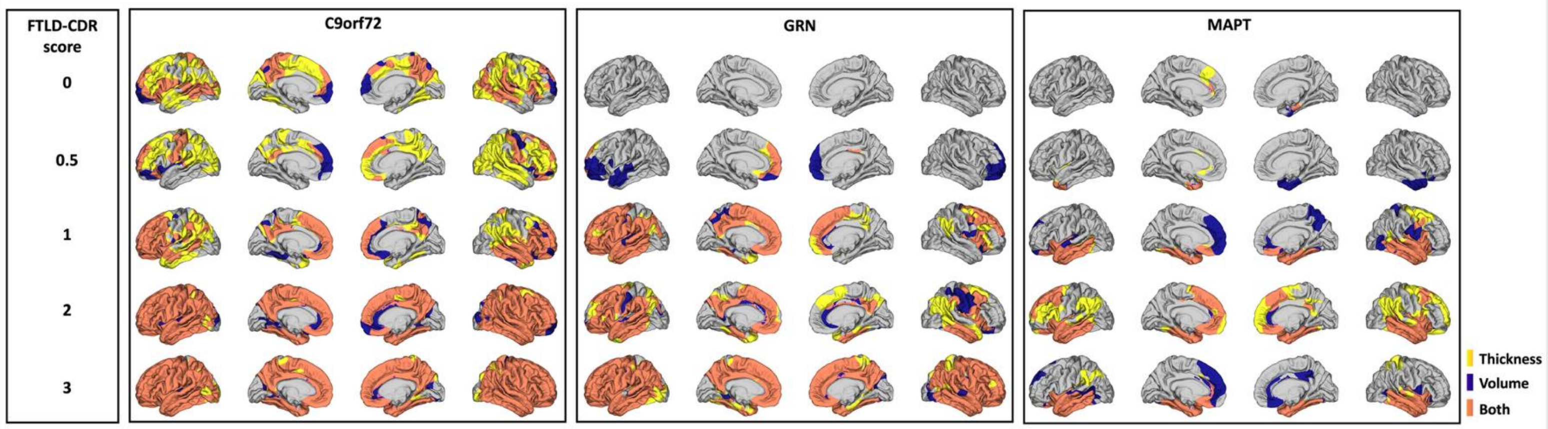

Supplementary Figure 3: Regions found to show significant differences for each FTD gene group and for varying levels of disease severity according to the FTLD-CDR score, compared to controls for either just volume, just thickness or both. p<0.003, Bonferroni corrected for multiple comparisons, cluster threshold = 0.01, FDR corrected, model adjusted for age, sex, years of education, site of acquisition and total intracranial volume.

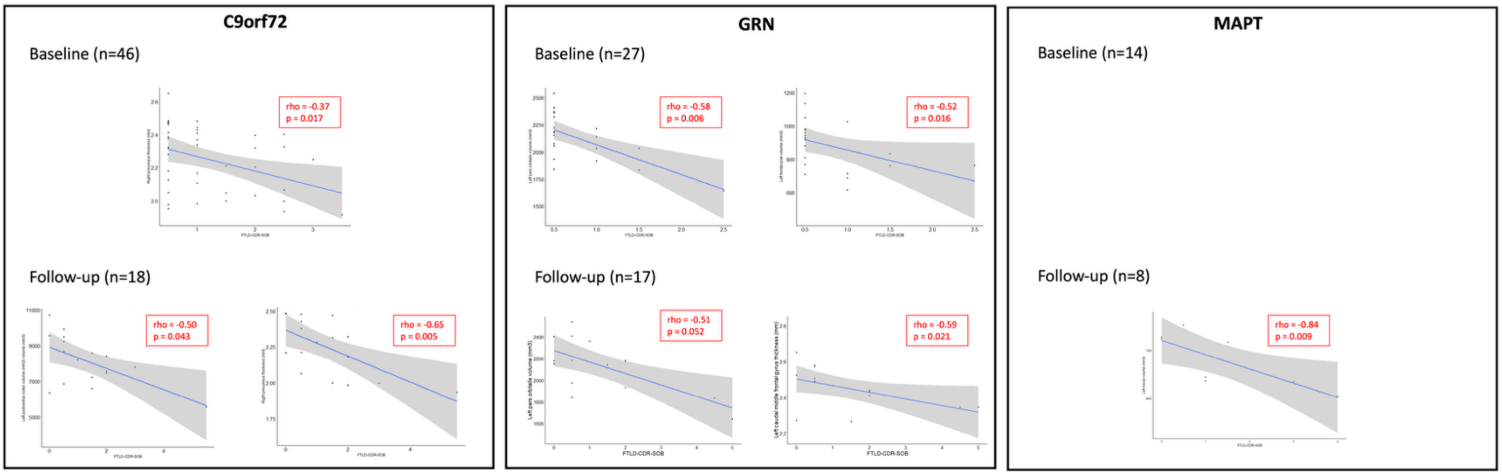

Supplementary Figure 4. Correlations between grey matter signatures and clinical severity as well as clinical progression in presymptomatic carriers, including cases with a clinical diagnosis.
